# The N-terminal dimerization domains of human and *Drosophila* CTCF have similar functionality

**DOI:** 10.1186/s13072-024-00534-w

**Published:** 2024-04-01

**Authors:** Sofia Kamalyan, Olga Kyrchanova, Natalia Klimenko, Valentin Babosha, Yulia Vasileva, Elena Belova, Dariya Fursenko, Oksana Maksimenko, Pavel Georgiev

**Affiliations:** 1 Department of the Control of Genetic Processes, Institute of Gene Biology, Russian Academy of Sciences, 34/5 Vavilov St, Moscow, 119334 Russia; 2 Center for Precision Genome Editing and Genetic Technologies for Biomedicine, Institute of Gene Biology, Russian Academy of Sciences, 34/5 Vavilov St, Moscow, 119334 Russia; 3 Skolkovo Institute of Science and Technology, Moscow, 121205 Russia

**Keywords:** Architectural proteins, Functional conservation, C2H2 zinc finger proteins, Bithorax, Dimerization domain

## Abstract

**Background:**

CTCF is highly likely to be the ancestor of proteins that contain large clusters of C2H2 zinc finger domains, and its conservation is observed across most bilaterian organisms. In mammals, CTCF is the primary architectural protein involved in organizing chromosome topology and mediating enhancer–promoter interactions over long distances. In *Drosophila*, CTCF (dCTCF) cooperates with other architectural proteins to establish long-range interactions and chromatin boundaries. CTCFs of various organisms contain an unstructured N-terminal dimerization domain (DD) and clusters comprising eleven zinc-finger domains of the C2H2 type. The *Drosophila* (dCTCF) and human (hCTCF) CTCFs share sequence homology in only five C2H2 domains that specifically bind to a conserved 15 bp motif.

**Results:**

Previously, we demonstrated that CTCFs from different organisms carry unstructured N-terminal dimerization domains (DDs) that lack sequence homology. Here we used the *CTCF*^*attP*(*mCh*)^ platform to introduce desired changes in the *Drosophila CTCF* gene and generated a series of transgenic lines expressing dCTCF with different variants of the N-terminal domain. Our findings revealed that the functionality of dCTCF is significantly affected by the deletion of the N-terminal DD. Additionally, we observed a strong impact on the binding of the dCTCF mutant to chromatin upon deletion of the DD. However, chromatin binding was restored in transgenic flies expressing a chimeric CTCF protein with the DD of hCTCF. Although the chimeric protein exhibited lower expression levels than those of the dCTCF variants, it efficiently bound to chromatin similarly to the wild type (wt) protein.

**Conclusions:**

Our findings suggest that one of the evolutionarily conserved functions of the unstructured N-terminal dimerization domain is to recruit dCTCF to its genomic sites in vivo.

**Supplementary Information:**

The online version contains supplementary material available at 10.1186/s13072-024-00534-w.

## Introduction

In higher eukaryotes, chromatin architecture is one of the essential determinants in the regulation of gene expression [1–7]. Existing models suggest that the CTCF protein, which contains a cluster of 11 Cys2-His2 zinc fingers (C2H2 domains), plays a key role in the organization of long-range interactions in mammals [8, 9]. Human CTCF protein (hCTCF) zinc fingers 3 to 7 specifically recognize a 15 bp long DNA motif [10]. It is assumed that in mammals, chromatin loops are formed by cohesin complexes whose movement along the chromatin fiber is blocked at the most stable CTCF binding sites [11–17]. A conserved ten amino acid residue stretch that binds to cohesin has been found in the N-terminal part of hCTCF [18]. On average, mammalian genomes contain 40,000–80,000 CTCF binding sites [19], which are often located at the boundaries of topologically associated domains (TADs), implying that CTCF facilitates long-range interactions between regulatory elements and participates in the organization of active promoters [20–22].

CTCF is the most studied representative of the largest group of DNA-binding transcription factors that contain clusters of at least five C2H2 domains [8, 23]. It has been demonstrated that several transcription factors within this group exhibit highly specific binding to 12–15 bp DNA motifs when comprising 4–5 C2H2 domains connected by specific 5 amino acid linkers [10, 24, 25]. C2H2 proteins belong to one of the most rapidly evolving groups of proteins [23], and CTCF is likely one of the progenitors of these proteins since it has been found in most studied bilaterian organisms [26]. However, within the primary sequence of CTCF, conservation is limited to the cohesin-binding motif [18] and five C2H2 domains that enable binding to specific DNA sequences [26]. Common structural features of CTCF in different bilaterians include the central location of the C2H2 cluster within the protein and the presence of an unstructured dimerizing domain (DD) at the N-terminus [27, 28]. In *Drosophila*, CTCF (dCTCF) does not play a dominant role in the organization of chromosome architecture [29–33] and binds to fewer than 1000 genomic sites, mainly in promoter regions [34, 35]. dCTCF inactivation affects the expression of genes involved in the functioning of nervous system (34) as well as the expression of Hox genes in the Bithorax complex [27, 34, 36–40], which represents an evolutionary conserved function of CTCF proteins [41–43]. In the Bithorax complex, dCTCF, in combination with Pita, Su(Hw), and additional yet unidentified architectural proteins, establishes the boundaries of regulatory domains that determine the expression of Hox genes [44, 45]. The Pita protein belongs to a large group of insect C2H2 proteins that feature zinc-finger associated domains (ZADs) at the N-terminus [46]. Structural analysis has shown that ZADs predominantly homodimerize [46, 47] and are necessary for organizing specific long-range interactions between the ZAD-C2H2 protein binding sites [48, 49]. In *Drosophila melanogaster*, over half of the 170 C2H2 proteins contain a ZAD domain [50], consistent with the proposed model of the cooperative contribution of C2H2 proteins to the organization of the *Drosophila* chromatin architecture [8, 51]. According to the model, multiple specific interactions between the homodimerizing domains of architectural C2H2 proteins play a key role in maintaining specific long-range interactions between regulatory elements [52]. Currently, the contribution of cohesin to the organization of *Drosophila* chromatin architecture remains unknown [7, 35, 53]. Interestingly, in mammals, specific long-range contacts between enhancers and promoters can also be maintained by homodimerization of the LDB1 protein, which is recruited simultaneously and independently to interacting enhancers and promoters [54]. Recent studies have also shown that, at certain loci, human CTCF establishes chromatin architecture together with other C2H2 proteins, such as MAZ or ZNF143 [55–57]. The ZNF143 protein can form chromatin loops independently of CTCF, although its interaction with the cohesin complex has not been confirmed [58, 59]. Meanwhile, the MAZ protein can interact with the cohesin complex, akin to CTCF [56]. Thus, accumulating experimental data support the idea that, similar to in *Drosophila*, a large group of C2Н2 proteins cooperatively establishes the architecture of mammalian chromosomes.

Here, we investigated the functional significance of individual regions within the 287 amino acid N-terminal domain of the *Drosophila* CTCF protein. Transgenic lines expressing mutant variants of the dCTCF protein tagged with the 3xHA epitope were generated for this purpose. Our results demonstrate that only the unstructured dimerization domain is functionally important. The mutant CTCF protein, lacking the dimerizing domain, loses its ability to effectively bind to a significant portion of its genomic target sites. However, the addition of the N-terminal domain from human CTCF restores the functional activity and specific DNA binding of the chimeric protein.

## Results

### Testing in vivo functions of the N-terminal regions of dCTCF

**Fig. 1:**
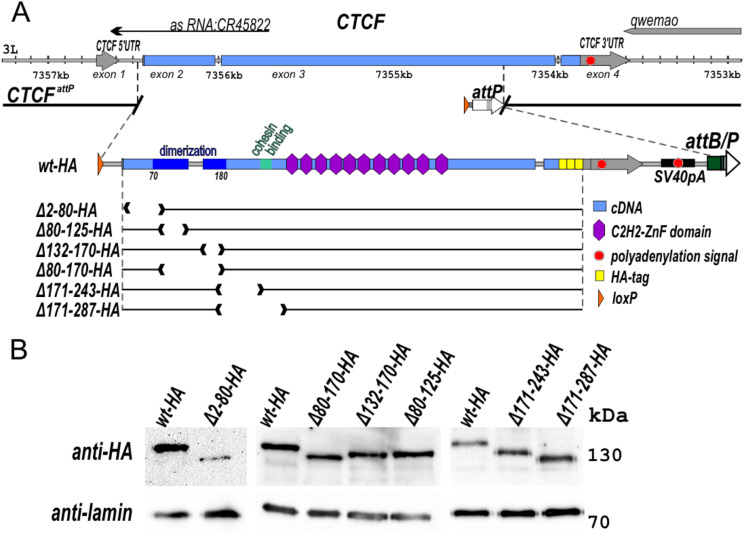
Mutations in the *CTCF* gene. (**A**) Schematic representation of dCTCF replacement. The untranslated regions of the *CTCF* gene are shown as grey arrows, and the coding region is marked with a blue line. The endpoint of the *CTCF*^*attP*(*mCh)*^platform is shown with breaks in the black line. The *attP, loxP*, and *attB* sites are shown as white, orange, and green arrows, respectively. Other designations are shown in the figure. The schematic representation of full-length restored *dCTCF-HA* (*wt-HA*) is shown below. The deletion variants of the *CTCF* gene are shown as black lines with breaks corresponding to the deleted domains. (**B**) Immunoblot analysis (6% SDS PAGE) of protein extracts prepared from adult flies of *dCTCF*^*wt*^*-HA (wt-HA), dCTCF*^*Δ2–80*^*-HA (Δ2-80-HA), dCTCF*^*Δ132–170*^*-HA (Δ132–170-HA), dCTCF*^*Δ80–170*^*-HA (Δ80–170-HA), dCTCF*^*Δ80–125*^*-HA (Δ80–125-HA), dCTCF*^*Δ171–243*^*-HA (Δ171–243-HA)*, and *dCTCF*^*Δ171–287*^*-HA (Δ171–287-HA)* lines with anti-HA and anti-lamin Dm0 (internal control) antibodies

The N-terminal region of dCTCF contains two conserved regions present in CTCF from different bilaterians (Fig. 1A): the unstructured dimerization domain [28] and a 10 amino acid residue stretch (244–254 aa) that binds to cohesin [35]. The N-terminal dimerization domain of dCTCF maps between 80 and 163 aa [27, 28].

We used a previously developed platform (*CTCF*^*attP*(*mCh*)^) to assess the functional role of the N-terminal region of dCTCF in vivo (Additional File 1: Fig. S1) [34]. This platform carries the substitution of a 2173-bp region of the *CTCF* gene, spanning from the first intron to the end of the third exon (3L:7,352,322–7,358,209, r6.52), with the *attP* site and *Act5C:mCherry* reporter flanked by *loxP* sites [34] (Fig. 1, Additional File 1: Fig. S1). The dCTCF replacement constructs included the *CTCF* genome region from the first intron to the fourth exon with a HA tag incorporated within the fourth exon, a polyadenylation signal from SV40, and the *white* reporter flanked by *loxP* sites (Additional File 1: Fig. S1). After the deletion of the reporter genes, only one *loxP* site remains in the first intron of the *CTCF* gene. As a result, we obtained *CTCF* alleles expressing wild type dCTCF-HA (CTCF^wt^-HA) and a group of dCTCF variants with the following deletions in the N-terminal region: 2–80 aa (Δ2–80-HA), 80–125 aa—proximal part of the dimerization domain (Δ80–125-HA), 132–170 aa—distal part of the dimerization domain (Δ132–170-HA), 80–170 aa—complete deletion of DD (Δ80–170-HA); 171–243 (Δ171–243-HA) and 171–287—including putative cohesin-interacting region (Δ171–287-HA) [18] (Fig. 1A). Western blotting analysis demonstrated that the dCTCF^Δ171–243^-HA, dCTCF^Δ171–287^-HA and dCTCF^Δ80–125^-HA variants are expressed at similar levels to dCTCF^wt^-HA. Despite the inaccuracy of the Western blot analysis, a slight decrease in expression can be observed for dCTCF^Δ132–170^-HA and dCTCF^Δ80–170^-HA in comparison to dCTCF^wt^-HA. Meanwhile, with the dCTCF^Δ2–80^-HA variant, the results of immunoblot analysis varied greatly: with anti-НА antibodies less protein was detected than dCTCF^wt^-HA, while at the same time, with anti-CTCF antibodies, the amount of protein became comparable to dCTCF^wt^-HA (Fig. 1B and Additional file 1: Fig. S2).

Unexpectedly, Western blot analysis also shows that dCTCF^wt^ (*y*^*1*^*w*^*1118*^ line) is expressed more strongly than dCTCF^wt^-HA. To confirm this finding, we analyzed the expression of dCTCF^wt^-HA and dCTCF^wt^ (*y*^*1*^*w*^*1118*^ line) in the cytoplasmic, nucleoplasmic, and chromatin fractions (Additional File 1: Fig. S3; Additional File 2). We found that the dCTCF^wt^-HA protein level was reduced in comparison with that of dCTCF^wt^. Comparison of the amount of dCTCF^wt^ protein in the *y*^*1*^*w*^*1118*^ line from one extraction procedure has demonstrated that most of it is associated with chromatin, while in the cytoplasm and nucleoplasm there is a smaller amount, but the protein is also detected (Additional File 1: Fig. S4). A previous study has shown that the 3xHA epitope leads to a decrease in the expression of tagged proteins in yeast for unknown reasons [60]. Further investigation is necessary to understand the mechanism underlying the decreased expression of proteins tagged with 3xHA.

**Fig. 2:**
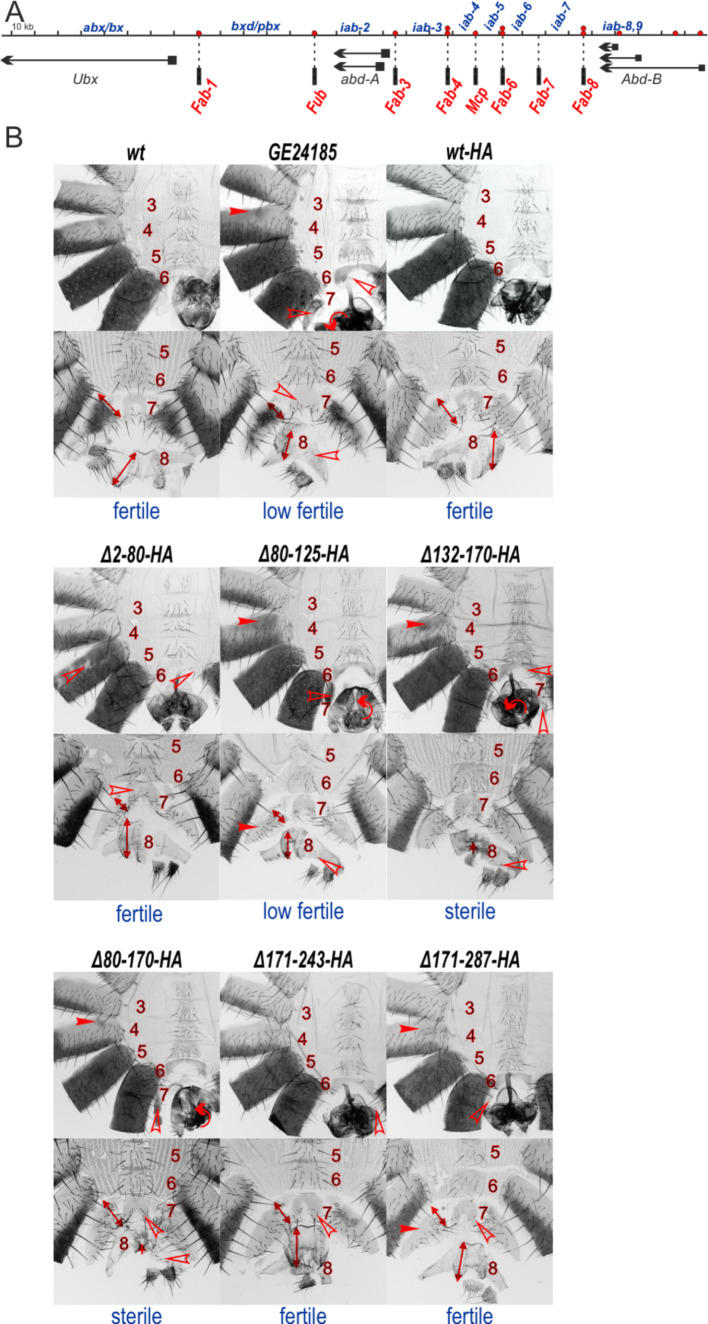
Morphology of the abdominal segments of the *dCTCF* mutants. (**A**) Scheme of BX-C (presented as a sequence coordinate line). The *Ubx*, *Abd-A*, and *Abd-B* transcripts are marked by horizontal black arrows. The boundary positions are indicated by vertical black bars with dotted lines. The mapped dCTCF-binding sites in the boundaries are indicated by red circles. (**B**) Morphology of the abdominal segments of the *dCTCF* mutants. Male (top) and female (bottom) abdominal cuticles are shown for lines expressing *wt* and mutant variants of dCTCF. The filled red arrowheads show morphological features indicative of transformations associated with increased or ectopic *Abd-B* expression. The empty red arrowheads indicate signs of transformations associated with decreased *Abd-B* expression relative to that in *wt*. The double-sided arrows indicate the size of the A7 tergite and female genitalia. Circular arrows indicate rotation of the male genitalia. Fertility was determined by the ability of males or females to produce offspring when crossed with males or females from the same line or *y*^*1*^*w*^*1118*^ line. “Fertile” means that the *CTCF* mutants produce offspring when crossed with each other. “Low fertile” means that the *CTCF* mutants cannot produce offspring when crossed with each other, but produce offspring when crossed with *CTCF*^*+*^ flies (*y*^*1*^*w*^*1118*^). “Sterile” means that the *CTCF* mutants cannot produce offspring when crossed with *CTCF*^*+*^ flies (*y*^*1*^*w*^*1118*^)

The null *CTCF* mutations mainly affect *Abd-B* expression in the *Drosophila* Bithorax complex (BX-C) [34, 36, 38]. The regulatory domains *iab-5*, *iab-6*, *iab-7*, and *iab-8,9* (Fig. 2A) control the gradual increase in the expression of *Abd-B* in parasegments (PS) PS10–PS14 that give rise to A5, A6, A7, and A8 (female) or A9 (male) adult segments, respectively [61]. The boundaries (Fig. 2A) provide functional autonomy to the *iab* domains during the regulation of *Abd-B* expression in the corresponding segments [37, 38, 44]. The dCTCF binding sites are located in the *Mcp* (1 site), *Fab-6* (2 sites), and *Fab-8* (2 sites) boundaries and in the promoter regions of *Abd-B*. In BX-C [44, 45, 62] and transgenic lines [63–65] dCTCF has been demonstrated to be essential for the activity of the *Mcp*, *Fab-6*, and *Fab-8* boundaries. Additionally, dCTCF binding sites are present in the promoters of many genes [34, 35, 65].

As an example of null *CTCF* mutants [34, 36, 38], *GE24185* males display held-out wings, partial transformation of A4 into A5 (ectopic pigmentation on A4 tergite), A6 into A5 (the presence of bristles on the A6 sternite), A7 into A6 (appearance of A7 tergite that is absent in *wild type* (*wt*)) and deformed genitalia (A8 (female), or A9 (male) segments) (Fig. 2B). *GE24185* flies are also characterized by delayed development and decreased viability. Both *GE24185* males and females are partially fertile and can produce progeny in crosses with *wt* flies. It has been shown that approximately 90% of eggs laid by homozygous *GE24185* parents remain unfertilized due to problems with the reproductive apparatus of the males and females, the development of which depends on proper *Abd-B* expression [36].

The expression of dCTCF^Δ2–80^-HA, dCTCF^Δ171–243^-HA, and dCTCF^Δ171–287^-HA mainly restored the viability, fertility and the *wt* phenotype of transgenic flies, similar to dCTCF^wt^-HA (Fig. 2B). Rare additional bristles were observed on the A6 sternite, indicating weak loss of function (LOF) of the *iab-6* domain. This finding suggests that the expression of these dCTCF deletion variants only weakly affects the expression of *Abd-B* in the A6 segment. A decrease in the expression of these dCTCF-HA variants compared to that of the dCTCF^wt^ protein could also be the reason for the observed weak mutant phenotype. These results imply that the deletions of 2–80 aa, 171–243 aa, and 171–287 aa do not significantly affect the functional activity of dCTCF, indicating that the potential cohesin-interacting motif [18] is not essential for dCTCF function (Fig. 2A). In contrast, the expression of dCTCF^Δ80–170^-HA (DD deletion) and dCTCF^Δ132–170^-HA (deletion of the distal part of the DD) led to a stronger mutant phenotype than the null *GE24185* mutation (Fig. 2A). While in *GE24185* females, the vaginal plates (A8) were only moderately reduced in size, in *dCTCF*^*Δ80–170*^*-HA* and *dCTCF*^*Δ132–170*^*-HA* females, the vaginal plates are absent or have partial transformation into abdominal segments. As a result, females expressing dCTCF^Δ80–170^-HA or dCTCF^Δ132–170^-HA are completely sterile. Similar to *GE24185* males, the deletion of DD in the dCTCF mutants leads to the appearance of pigmented spots on the A4 tergite, which is explained by the inability of the *Mcp* boundary to effectively block crosstalk between the *iab-4* and *iab-5* enhancers, resulting in ectopic *Abd-B* expression in the A4 segment. However, in contrast to *GE24185* the *dCTCF*^*Δ132–170*^*-HA* males are sterile. The deletion of the 80–125 aa (the proximal part of DD) from dCTCF had a moderate effect on dCTCF functionality, and the phenotype of mutant flies was similar to *GE24185* flies: several bristles on the A6 sternite, the male genitalia rotated and extruded, female genitalia reduced in size. The degree of genital deformation and the level of protein in the maternal gonads limit fertility of the *dCTCF*^*Δ80–125*^, as in *GE24185* (Fig. 2A). Thus, the N-terminal DD (80–170 aa) is key for dCTCF functions in vivo.

**Fig. 3:**
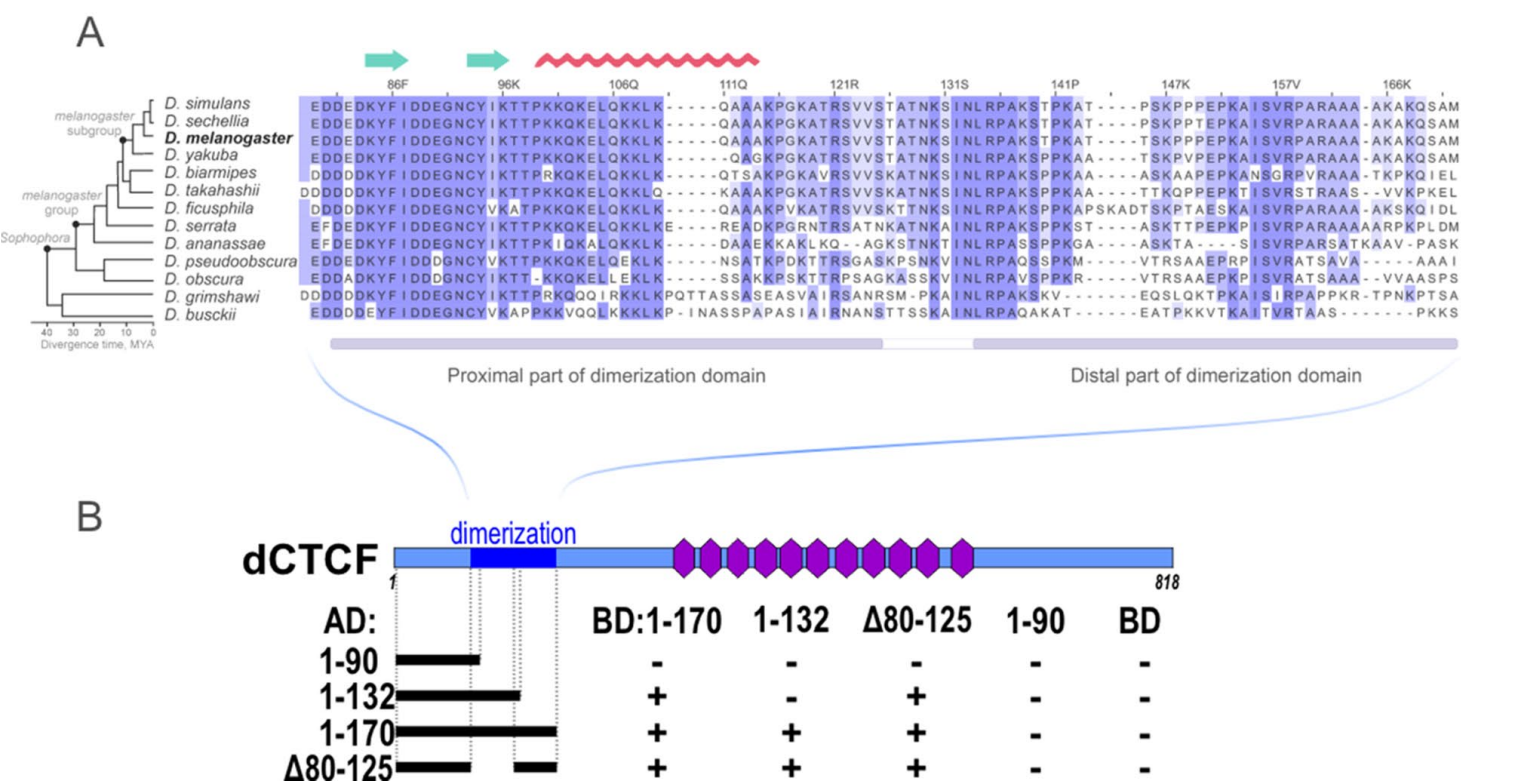
Results of the Y2H assay of the dimerization activity of the proximal and distal parts of DD from dCTCF. (**A**) Multiple sequence alignment of the N-terminal DD regions from different *Drosophila* species. Color intensities indicate percentage identity. Secondary structures were predicted with AlphaFold (https://alphafold.ebi.ac.uk/): green arrows represent putative beta sheets, and the red line represents the alpha helix. (**B**) AD represents the GAL4 activation domain, and BD stands for the GAL4 DNA binding domain. The ‘+’ interaction indicates the ability of yeast to grow on assay plates without histidine

We investigated the ability of the proximal and distal parts of the domain to dimerize in the yeast two-hybrid system to confirm the modular structure of the DD (Fig. 3). Our findings confirmed that the first ninety amino acids of the N-terminus (dCTCF 1–90) are incapable of homodimerization and interaction with the full fragment, dCTCF 1–170. Although dCTCF 1–132 cannot homodimerize, it can interact with dCTCF 1–170 or dCTCF Δ80–125. In contrast, dCTCF 1–170 can homodimerize and interact with dCTCF Δ80–125, whereas dCTCF Δ80–125 itself can undergo homodimerization. These results demonstrate that the distal 132–170 aa region is the core part of the domain that can homodimerize and interact with the proximal 80–125 aa region.

### The N-terminal dimerization domain is essential for dCTCF binding

One of the functional roles of the DD may involve facilitating the efficient chromatin binding of dCTCF. We performed a genome-wide chromatin immunoprecipitation followed by high-throughput sequencing (ChIP-seq) analysis to evaluate the binding of dCTCF^wt^-HA and dCTCF^Δ132–170^-HA in adult flies. Although embryos are best suited for ChIP-seq analysis, we used 3-day-old flies because dCTCF^Δ132–170^-HA homozygotes are sterile. dCTCF is expressed in oocytes and exhibits a maternal effect, which may influence the results of association with chromatin of the dCTCF^Δ132–170^-HA mutant. We collected chromatin samples from three-day-old flies and performed ChIP using antibodies against the 3xHA epitope followed by Illumina high-throughput sequencing.

**Fig. 4:**
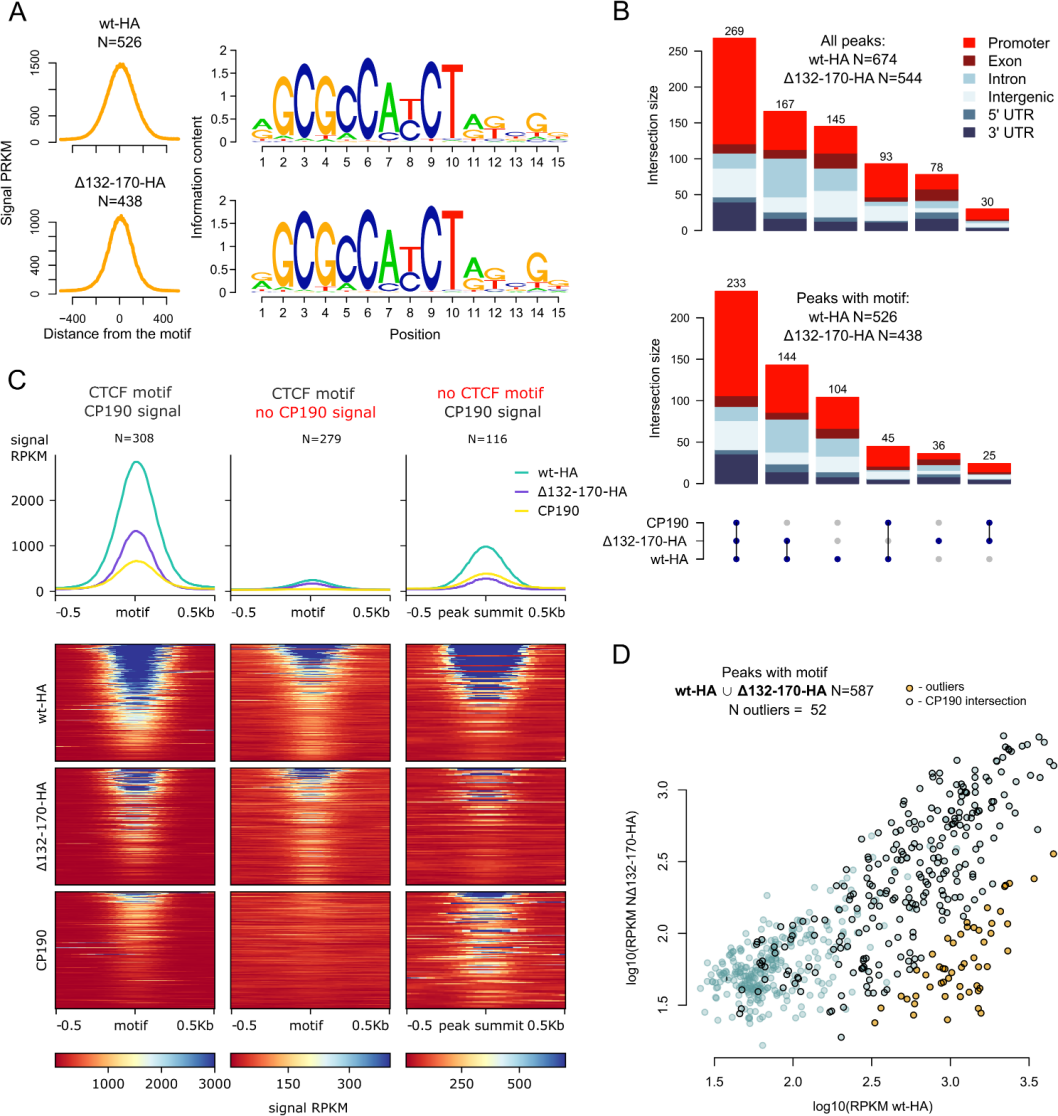
Comparison of dCTCF binding in *CTCF*^*wt*^*-HA* and *CTCF*^*Δ132–170*^*-HA* adult flies. (**A**) Average signal (RPKM) of ChIP-seq peaks with DNA binding motif (left) and motif logos (right) for CTCF^wt^-HA (wt-HA) and CTCF^Δ132–170^-HA (Δ132–170-HA). The motifs associated with the peaks in both datasets are the same. (**B**) UpSet plot showing an overlap of CP190 binding sites in the *y*^*1*^*w*^*1118*^ line and dCTCF binding sites in *CTCF*^*wt*^*-HA (wt-HA)* and *CTCF*^*Δ132–170*^*-HA (Δ132–170-HA)* lines considering all dCTCF sites (top) and only sites with the binding motif (bottom). The bars are colored according to the distribution of the genomic elements in the corresponding regions. Only regions with dCTCF binding sites are shown. (**C**) Average signals (RPKM) (top) and signal heatmaps (bottom) for CP190 peaks in the *y*^*1*^*w*^*1118*^ line and dCTCF peaks in the *CTCF*^*wt*^*-HA (wt-HA)* and *CTCF*^*Δ132–170*^*-HA (Δ132–170-HA)* lines. Three different sets of peaks are displayed (columns): a combined set of dCTCF binding sites with the binding motif (both wt-HA and Δ132–170-HA) overlapping with CP190 binding sites (denoted as “dCTCF motif; CP190 signal”); a combined set of dCTCF binding sites with the motif and not overlapping with CP190 binding sites (denoted as “dCTCF motif; no CP190 signal”); a combined set of dCTCF binding sites without the motif but overlapping with CP190 binding sites (denoted as “no dCTCF motif; CP190 signal”). The “dCTCF motif; CP190 signal” and “dCTCF motif; no CP190 signal” sets were motif-centered, and “no CTCF motif; CP190 signal” set was centered on the peak summit. (**D**) Comparison of dCTCF signal (RPKM) in the *CTCF*^*wt*^*-HA (wt-HA)* and *CTCF*^*Δ132–170*^*-HA (Δ132–170)* lines in the combined set of dCTCF peaks with the binding motif. Outliers were detected using linear regression (see Methods). Regions overlapping with CP190 binding sites are circled in black

Comparing the binding profiles, we identified 674 peaks for dCTCF^wt^-HA and 544 peaks for dCTCF^Δ132–170^-HA. Both dCTCF^wt^-HA and dCTCF^Δ132–170^-HA bound to the same DNA binding motif in 526 and 438 peaks, respectively (Fig. 4A). Among these motif-confirmed peaks, 233 (with motif/269 total) peaks overlapping both dCTCF^Δ132–170^-HA and dCTCF^wt^-HA colocalized with CP190 (Fig. 4B). Over 50% of these colocalizing peaks were found in promoter regions. Additionally, we observed relatively small groups of sites colocalized with CP190 that were exclusively bound by either dCTCF^wt^-HA (45 with motif/total 93) or dCTCF^Δ132–170^-HA (24 with motif/total 30). There were a significant number of peaks that did not overlap with the CP190 peaks and where dCTCF^Δ132–170^-HA and dCTCF^wt^-HA bound the same sites (143 peaks with a motif out of a total of 166). Sites bound exclusively by dCTCF^Δ132–170^-HA had a significantly lower frequency of binding motif occurrence compared to sites overlapping with CP190 or dCTCF^wt^-HA (*p* = 1.5·10^− 18^ chi-squared test, *N* = 544). They were also significantly less frequent in promoters (*p* = 0.0013, chi-squared test, *N* = 544)) (Fig. 4B). In terms of signal levels, CP190-colocalizing peaks demonstrated strong binding by both dCTCF^wt^-HA and dCTCF^Δ132–170^-HA, whereas their binding to peaks lacking the CP190 signal was weaker (Fig. 4C, *p* = 3.4·10^− 77^ for dCTCF^wt^-HA and *p* = 3.1·10^− 35^ for dCTCF^Δ132–170^-HA, two-sided Mann-Whitney U test for peaks with motif, N1 = 308, N2 = 279). These findings indicate that CP190 is associated with most robust dCTCF binding sites. In addition, the average signal intensity of dCTCF^Δ132–170^-HA peaks with the motif was reduced at least two-fold compared to that of the dCTCF^wt^-HA peaks (*p* = 7.5·10^− 60^ two-sided Wilcoxon rank-sum test for peaks with motif, *N* = 587 × 2). Notably, a significantly reduced signal in dCTCF^Δ132–170^-HA binding compared to that with dCTCF^wt^-HA was observed in 52 peaks associated with CP190 (Fig. 4D, *p* < 0.05, see Methods). These results suggest that the N-terminal dimerization domain is essential for the efficient binding of dCTCF to a substantial number of its sites.

### The N-terminal domain of hCTCF can functionally substitute the DD of dCTCF

Since the human N-terminal domain also contains an unstructured dimerization domain [28] (Additional File 1: Fig. S5), we hypothesized that the hCTCF N-terminal domain could function in *Drosophila* despite the lack of sequence homology with the DD of dCTCF. To test this, we generated a construct expressing a chimeric protein dCTCF^hN^ in which the 265 amino acid N-terminus of hCTCF was fused to the dCTCF without the DD (dCTCF^Δ80–170^). To compare the ability of the N-terminal domains of dCTCF^wt^, dCTCF^Δ80–170^ and dCTCF^hN^ to dimerize, we fused the tested N-terminal domains to FLAG or HA epitopes in expression vectors. The same N-terminal domains, fused to either FLAG or HA, were coexpressed in S2 *Drosophila* cells, and their ability to interact was assessed by co-immunoprecipitation (Additional File 1: Fig. S6). We have confirmed that the N-terminal domain of dCTCF^wt^, as well as of dCTCF^hN^, is able to oligomerize, whereas that of dCTCF^Δ80–170^ is not.

**Fig. 5:**
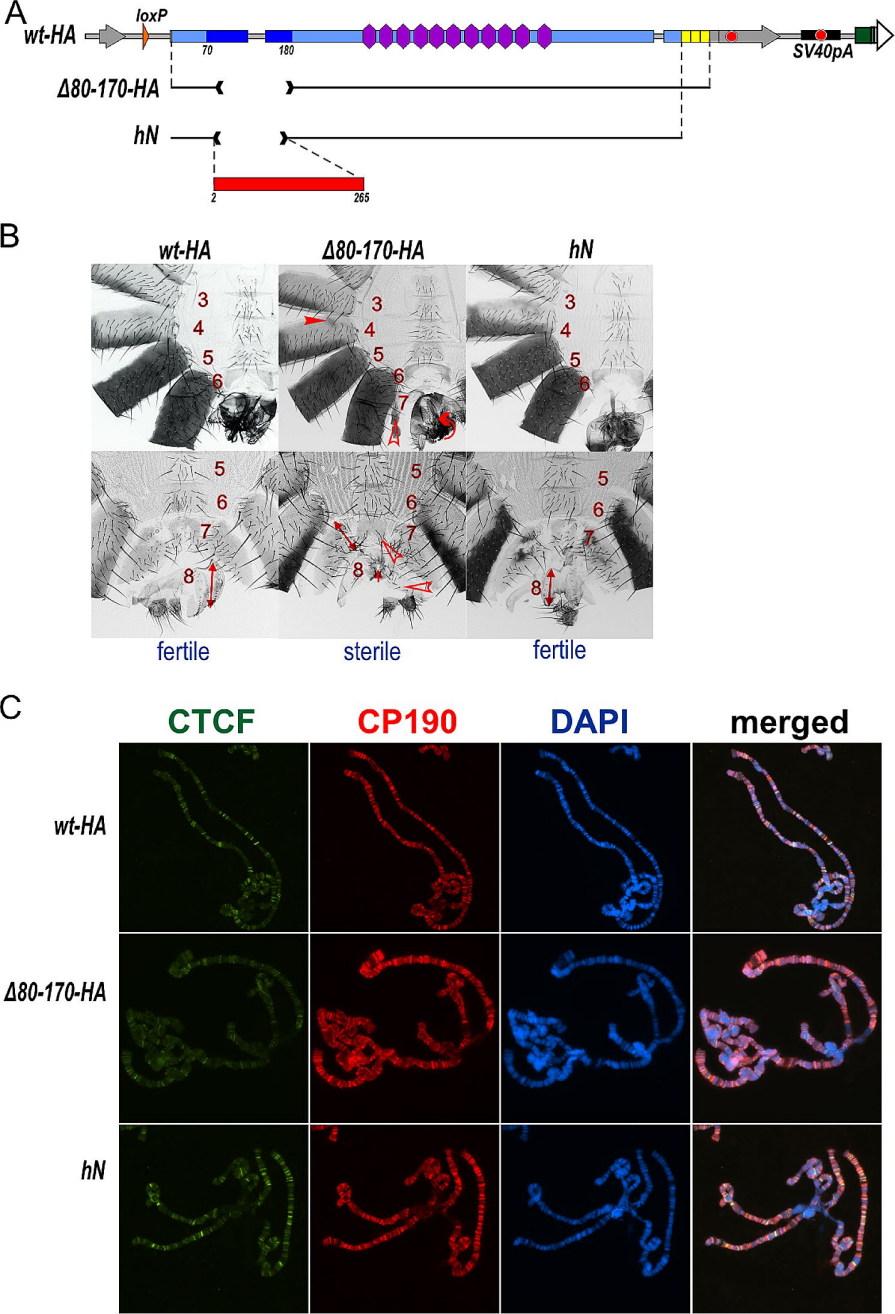
The dimerization domain from hCTCF can functionally replace the DD in dCTCF. (**A**) Schematic representation of *dCTCF*^*wt*^*(wt-HA)* and its mutant variants *dCTCF*^*Δ80–170*^*-HA (Δ80–170-HA)* and *dCTCF*^*hN*^*(hN)*. (**B**) Morphology of the abdominal segments of the lines with *dCTCF* mutations. The designations correspond to those shown in Figs. 1 and 2. (**C**) Localization of dCTCF^wt^, dCTCF^Δ80–170^-HA, and dCTCF^hN^ in the polytene chromosomes from third instar female larvae of respective fly lines. The panels show the results of immunostaining of dCTCF variants (with rabbit anti-dCTCF_C antibodies) and CP190 (rat anti-CP190 antibody). DNA was stained with DAPI (blue). Scale bar is 20 μm

Next, the transgenic line expressing CTCF^hN^-HA was created. We were unable to detect any protein by Western blot analysis. To enhance the expression of the chimeric protein, we obtained a transgenic line expressing CTCF^hN^ without the HA tag (Fig. 5A).

**Fig. 6:**
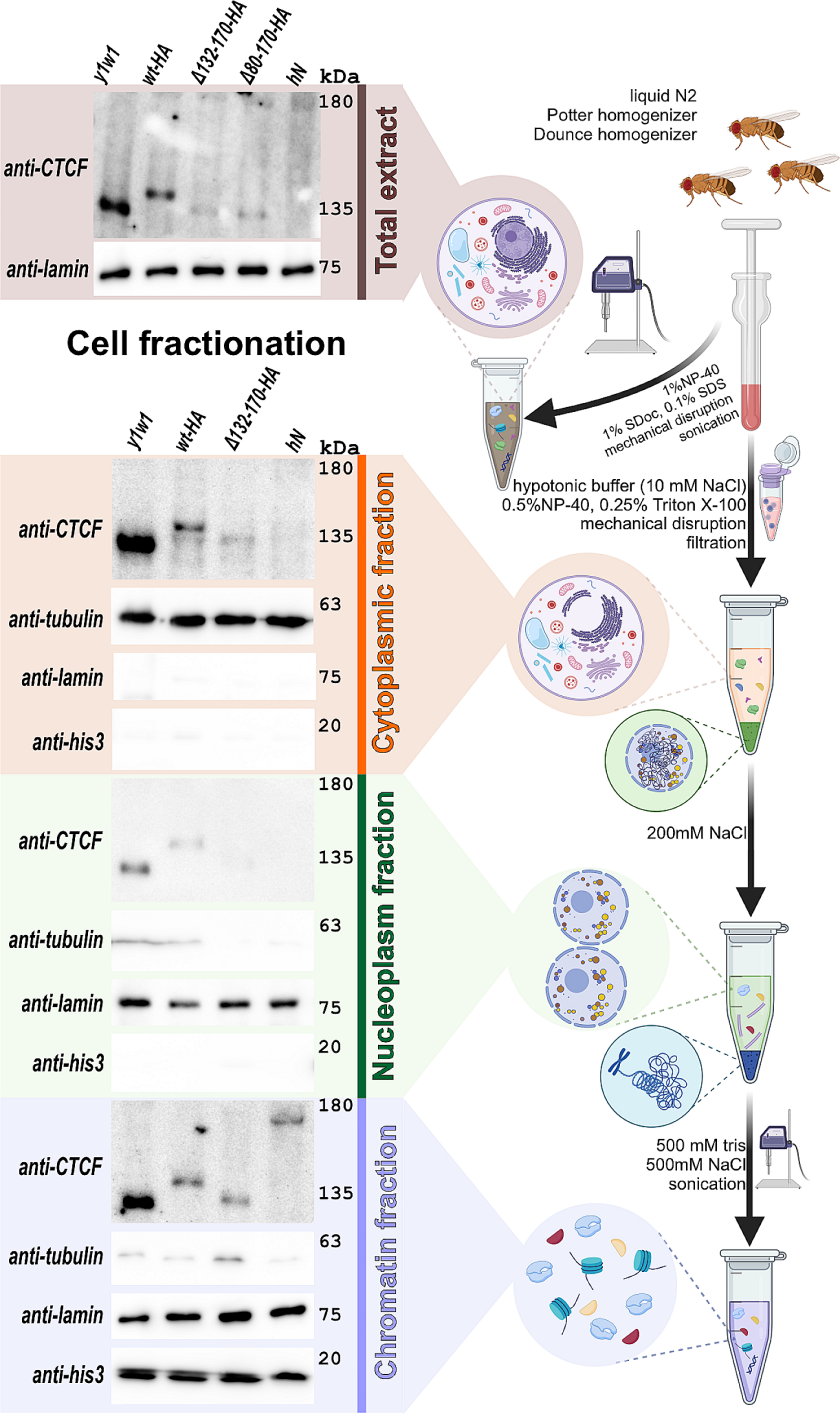
Immunoblot analysis of total extract and cytoplasmic, nucleoplasmic, chromatin fractions prepared from two-day-old adult males of *y*^*1*^*w*^*1118*^ *(y1w1), dCTCF*^*wt*^*-HA (wt-HA), dCTCF*^*Δ132–170*^*-HA (Δ132–170-HA), dCTCF*^*Δ80–170*^*-HA (Δ80–170-HA), and dCTCF*^*hN*^ *(hN)* lines. The total extract and cytoplasmic, nucleoplasmic, and chromatin fractions were prepared as described in the Materials and Methods section and briefly explained in the right panel of the figure. Blots were stained with anti-dCTCF_C antibodies and control antibodies against tubulin (cytoplasmic marker), lamin (nuclear marker), and histone H3 (his3, chromatin marker)

We used antibodies against the C-terminal domain of dCTCF [34] to detect the dCTCF variants. Again, we were unable to detect dCTCF^hN^ in the total protein extract by Western blotting (Fig. 6, Additional File 3). We have found that CTCF^hN^ was expressed at very low levels due to incorrect splicing of the first intron of the transgene (Additional File 1: Fig. S7). Also, the expression levels of dCTCF^Δ80–170^-HA and dCTCF^Δ132–170^-HA were reduced compared to the levels of dCTCF^wt^-HA. Next, we compared the expression levels of dCTCF^wt^-HA, dCTCF^Δ132–170^-HA, and dCTCF^hN^ in the cytoplasmic, nucleoplasmic, and chromatin fractions (Fig. 6, Additional File 3). The band corresponding to dCTCF^hN^ was not detected in the cytoplasmic and nucleoplasmic fractions. However, all three dCTCF variants (dCTCF^wt^-HA, dCTCF^Δ132–170^-HA, and dCTCF^hN^) were present at comparable levels in the chromatin fraction.

Despite its low expression, dCTCF^hN^ largely restored the *wild type* phenotype as the dCTCF^wt^-HA (Fig. 5B). Furthermore, we examined polytene chromosomes from third instar larvae expressing the dCTCF variants to assess their binding patterns. The C-terminal dCTCF antibodies stained over one hundred interband regions of the polytene chromosomes of larvae expressing dCTCF^wt^-HA (Fig. 5C). A similar pattern of polytene chromosomes was observed in larvae expressing dCTCF^hN^. In larvae expressing dCTCF^Δ80–170^-HA, however, the fluorescence intensity was decreased, and some bands were absent. These results indicate that the antibodies effectively recognize dCTCF sites in polytene chromosomes and that dCTCF^hN^ binds to chromatin with the same efficiency as dCTCF^wt^-HA. Conversely, the binding efficiency of dCTCF^Δ80–170^-HA is reduced (Fig. 5C).

**Fig. 7:**
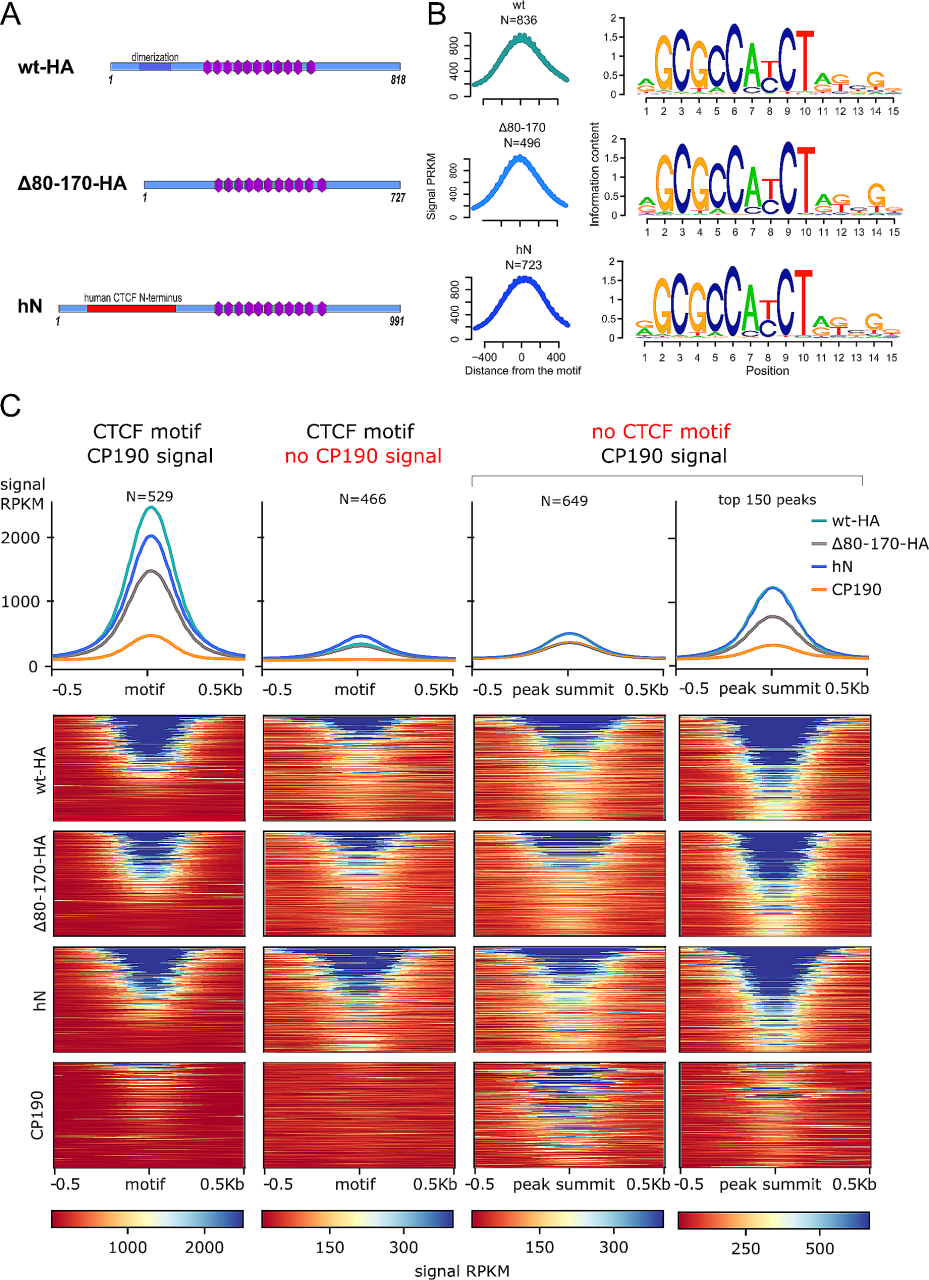
Comparison of dCTCF^wt^-HA (wt), dCTCF^Δ80–170^-HA(Δ80–170) and dCTCF^hN^ (hN) binding in adult flies. (**A**) Average signal (RPKM) of ChIP-seq peaks with motif (left) and motif logo (right) for dCTCF protein (anti-dCTCF_C antibodies) in lines expressing dCTCF^wt^-HA (wt), dCTCF^Δ80–170^-HA (Δ80–170) and CTCF^hN^ (hN). The motifs associated with the peaks are the same in all three datasets. (**B**) Average signal (RPKM) (top) and signal heatmaps (bottom) for CP190 in the *y*^*1*^*w*^*1118*^ line and dCTCF in the *wt-HA*, *Δ80–170-HA*, and *hN* lines. Different sets of peaks for all three lines (*wt-HA, Δ80–170-HA*, and *hN*) are shown (columns): a combined set of dCTCF binding sites with the binding motif overlapping with CP190 binding sites (denoted as “dCTCF motif; CP190 signal”); a combined set of dCTCF binding sites with the motif and not overlapping with CP190 binding sites (denoted as “dCTCF motif; no CP190 signal”); a combined set of dCTCF binding sites without the motif but overlapping with CP190 binding sites (denoted as “no dCTCF motif; CP190 signal”). The “dCTCF motif; CP190 signal” and “dCTCF motif; no CP190 signal” sets were motif-centered, and “no CTCF motif; CP190 signal” set was centered on the peak summit

**Fig. 8:**
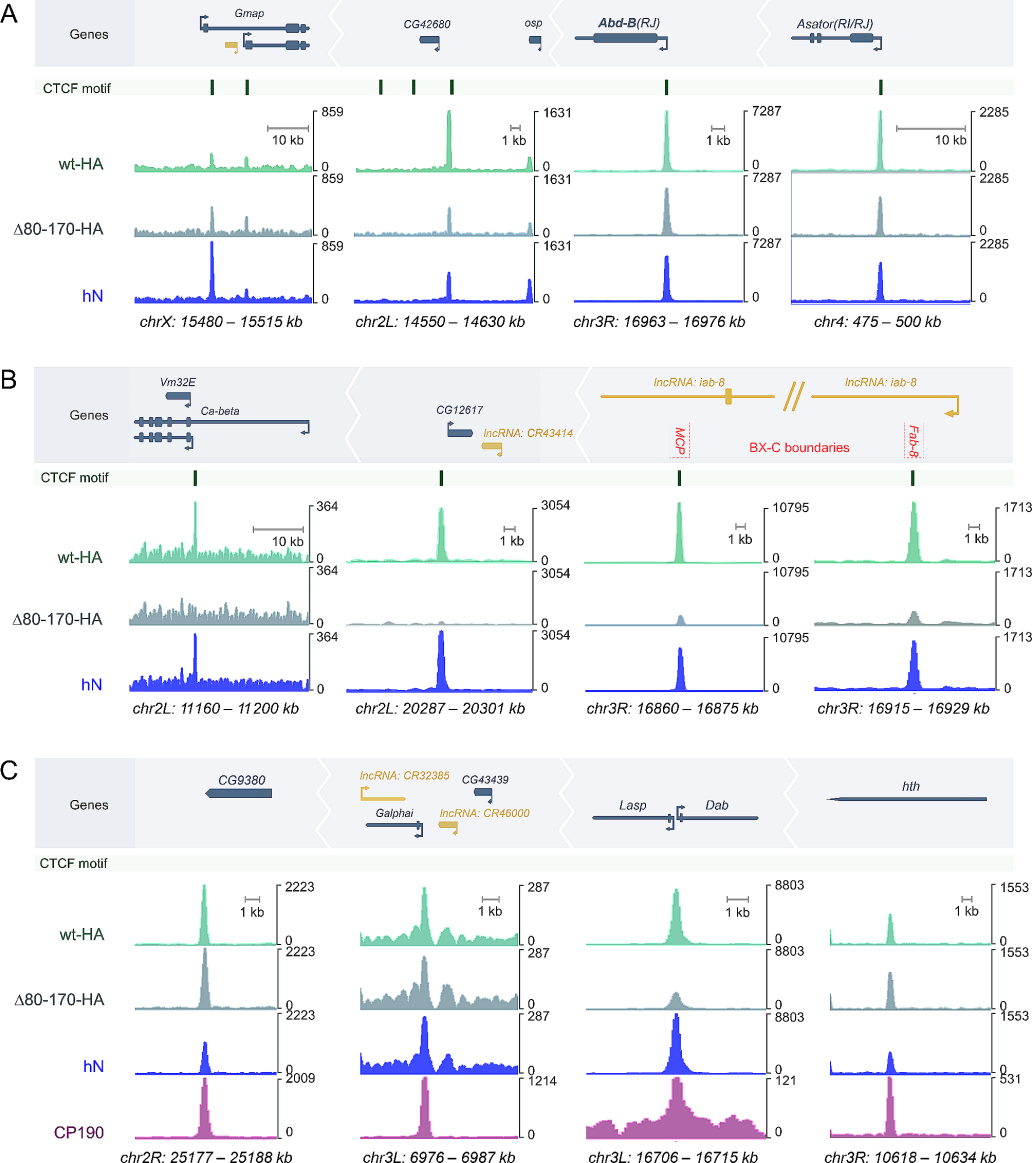
Genome browser tracks for representative regions with occupancy of dCTCF^wt^HA, dCTCF^Δ80–170^-HA, dCTCF^hN^. The dCTCF motif track is shown for convenience. Protein-coding genes are depicted in dark blue, lncRNA-coding genes are shown in yellow, and arrowheads indicate promoters. (**A**) Regions with CTCF^wt^HA, CTCF^d80–170^-HA, and dCTCF^hN^ occupancy, with peaks confirmed by motifs. (**B**) Regions lacking CTCF^d80–170^-HA binding with peaks confirmed by motifs. Red dashed lines mark the boundaries of the regulatory *iab* domains. (**C**) Regions with CTCF^wt^HA, CTCF^d80–170^-HA, dCTCF^hN^ occupancy, and CP190 enrichment, lacking the dCTCF DNA binding motif

Next, we investigated whether the human N-terminal domain restores the efficiency of dCTCF binding to genomic sites. We performed genome-wide ChIP-seq analysis with antibodies against the C-terminus of dCTCF to assess the binding of dCTCF^wt^-HA, dCTCF^Δ80–170^-HA, and dCTCF^hN^ in adult flies. We identified a total of 836 peaks for dCTCF^wt^-HA, 496 peaks for dCTCF^Δ80–170^-HA, and 723 peaks for dCTCF^hN^, all containing the same motif (Fig. 7A). Again, peaks associated with CP190 were significantly stronger (Fig. 7B, *p* = 6.0·10^− 79^ for dCTCF^wt^-HA, *p* = 2.1·10^− 68^ for dCTCF^Δ80–170^-HA and *p* = 3.2·10^− 52^ for dCTCF^hN^, two-sided Mann-Whitney U test for peaks with motif, N1 = 529, N2 = 466). The average signal of dCTCF^hN^ was only slightly weaker than that of dCTCF^wt^-HA. In contrast, the average signal of dCTCF^Δ80–170^-HA was decreased by more than half in comparison with that of dCTCF^wt^-HA and dCTCF^hN^ (Fig. 7B, comparison between dCTCF^Δ80–170^-HA and dCTCF^hN^ signals: *p* = 6.5·10^− 66^ for peaks with motif *N* = 995 × 2). Interestingly, a small group of promoters, including an *Abd-B* promoter, demonstrated equal binding efficiency for all three dCTCF variants (Fig. 8A), indicating that DD is not essential for dCTCF binding in some genomic regions. However, at the most robust dCTCF/CP190 sites, including the *Fab-8* and *Mcp* boundaries, both dCTCF^wt^-HA and dCTCF^hN^ showed increased binding in comparison with that of dCTCF^Δ80–170^-HA (Fig. 8B). At these specific genomic sites, the human dimerization domain compensates for the deletion of the DD in dCTCF and restores binding of the dCTCF^hN^ protein to its target sites.

We observed weak average signals in two sets of peaks: one with the CTCF motif but without the CP190 signal, and the other without the CTCF motif but with the CP190 signal (Fig. 7B). Interestingly, all dCTCF variants displayed similar average binding efficiency in both sets of peaks. High-signal CP190 sites colocalizing with weak dCTCF genomic sites were mainly located in the promoter region, 5’ untranslated region (5’UTR), 3’ untranslated region (3’UTR), and within the gene (Fig. 8C). Notably, the deletion of the DD did not appear to affect dCTCF binding to these genomic regions. Thus, the DD is critical for dCTCF binding to strong CP190-dependent sites where dCTCF plays an important functional role, such as the *Mcp* and *Fab-8* boundaries.

## Discussion

Our analysis of N-terminal deletions of the dCTCF protein revealed that the dimerization domain plays a crucial role in the activity of dCTCF in vivo. The DD is required for the efficient binding of dCTCF to a set of genomic sites, including boundaries in the Bithorax complex. Although the unstructured dimerization domains of human and *Drosophila* CTCF proteins lack sequence homology [28], they represent a prominent feature of CTCF proteins alongside the highly conserved C2H2 domains that mediate binding to a similar 15 bp DNA motif across different species [26]. Unlike the *Drosophila *N-terminal domain, the human N-terminal domain does not dimerize when expressed in bacteria, which may be due to disruption of its correct folding [28]. This is the reason for conflicting results about the ability of the N-terminal domain of human CTCF protein to homodimerize [28, 66, 67]. However, the results presented here and in a previous study [28] show that the N-terminal domain of human CTCF dimerizes when it is expressed in yeast and *Drosophila* S2 cells. In this study, we demonstrated the functional equivalence of the dimerization domains between *Drosophila* and human CTCF proteins. Despite its low expression level, the chimeric dCTCF^hN^ protein efficiently bound to genomic sites comparably to dCTCF^wt^-HA, demonstrating fully restored functional activity. Given the lack of homology between the dimerization domains of human and *Drosophila* CTCF proteins, it is likely that the dimerization activity, rather than the ability of this domain to interact with partner proteins, plays a significant role in the functionality of dCTCF.

Our findings suggest that the 132–170 amino acid region is the core part of the DD, enabling homodimerization and interaction with the 80–132 amino acid region. The flexible organization of unstructured dimerization domains contributes to their rapid sequence variability during evolution, facilitating functional improvement or acquisition of new functions. We previously observed a similar pattern in the multifunctional zinc finger transcription factor CLAMP, which also contains an intrinsically disordered N-terminal domain consisting of two parts both capable of homo- and heterodimerization [68]. However, in contrast to the low conservation of the CTCF DD sequence, even among *Drosophila* species, the N-terminal dimerization domain of CLAMP is conserved across most insects.

DD is likely involved in the formation of local chromatin loops between dCTCF sites. However, proving this through Hi-C or Micro-C experiments in flies is challenging. Even in early embryos [69] and cell lines [70] with highly homogeneous chromatin, complete inactivation of dCTCF does not significantly affect chromatin architecture. This effect may be attributed to the collaborative role of dCTCF with other architectural proteins in forming regulatory elements and establishing chromatin loops [7, 52]. For example, dCTCF forms the boundaries of regulatory domains in the BX-C together with Pita, Su(Hw), and other yet-unidentified architectural proteins [49]. Recent studies have shown that mammalian CTCF also forms topologically associating domain boundaries in cooperation with at least several other proteins, including some that interact with cohesin [55–57, 71]. One of the prominent roles of dCTCF is its involvement in long-range interactions between the *iab* enhancers and the *Abd-B* promoter and in the establishment of insulators between adjacent *iab* domains. For example, in dCTCF mutants, ectopic activation of *Abd-B* in the A4 segment implies that reduced dCTCF binding affects the insulator function of the *Mcp* boundary located between the *iab-4* and *iab-5* domains (Fig. 1A).

In the present study, most robust dCTCF binding sites colocalized with CP190 peaks in chromatin samples from adult flies. In adult flies, binding of dCTCF variants was examined in an average mixture of a large number of different cell types. It is possible that in some cell types dCTCF loses its ability to bind to regulatory elements, which become inactive. Because CP190 binds preferentially to the promoters of housekeeping genes, dCTCF binding is maintained at high levels at such genomic sites. Previous studies have demonstrated that the C-terminal region spanning amino acids 705 to 733 of the dCTCF protein interacts with the BTB and M domains of the CP190 protein [34, 72]. In flies expressing the mutant dCTCF^Δ705–733^ protein, the efficiency of CP190 binding remains largely unaffected [34], consistent with the model that each regulatory element, along with dCTCF, is associated with other architectural proteins that also interact with CP190 [7, 52]. Thus, the robust dCTCF/CP190 sites are bound by groups of architectural proteins that recruit CP190. Interestingly, while dCTCF^Δ705–733^ binding is not impaired in comparison with that of dCTCF^wt^ [73], several studies have shown that CP190 inactivation results in reduced dCTCF binding [38, 74]. This observation can be explained by the essential role of CP190 in the formation of open chromatin regions [73, 75–78], which likely facilitates the binding of architectural C2H2 proteins to chromatin.

Our results also suggest that dCTCF can bind to genomic sites lacking the consensus motif independent of the presence of the DD. However, the binding of dCTCF to such motif-lacking sites is relatively weak and could be mediated by direct interactions with unknown DNA-binding architectural proteins and transcription factors. Further studies are required to understand the mechanisms underlying the motif-independent binding of dCTCF.

## Materials and methods

### Fly crosses and generation of transgenic lines

*Drosophila* strains were maintained on standard medium at 25 °C and 50–60% humidity. A previously obtained fly line with the landing platform (*CTCF*^*attP(mCh)*^) [34] was used for the insertion of transgenic constructs using the φC31-mediated site-specific integration system [79]. The constructs were assembled based on the *pBluescriptSK* vector and contained genetic elements in the following order: [*mini-white*]-[*loxP*]-[modified *CTCF*]-[*SV40polyA*]-[*attB*] (Additional File 1: Fig. S1). Mutations in the protein-coding region were generated by overlap extension polymerase chain reaction (PCR) using the primers listed in Additional File 4 and were verified by sequencing. Successful genomic integration events were visualized through the expression of the *mini-white* reporter in the eyes. The *mini-white* and *Act5C:mCherry* reporters were further excised by Cre-mediated recombination between the *lox* sites. All crosses were conducted using the TM6 balancer. Fertility was determined by the ability of males or females to produce offspring when crossed with males or females from the same line or *y*^*1*^*w*^*1118*^ line. “Fertile” means that the CTCF mutants produce offspring when crossed with each other. “Low fertile” means that the CTCF mutants cannot produce offspring when crossed with each other, but produce offspring when crossed with CTCF^+^ flies (*y*^*1*^*w*^*1118*^). “Sterile” means that the CTCF mutants cannot produce offspring when crossed with CTCF^+^ flies (*y*^*1*^*w*^*1118*^). Details of the crosses, the primers used for the genetic analysis, and all stocks are available upon request.

### Cuticle preparations

Three-day-old adult flies were collected in 1.5 ml tubes and stored in 70% ethanol for at least 1 day. The ethanol was then replaced with 10% KOH, and the flies were heated at 70 °C for 1 h. After heating, the flies were washed twice with dH2O and heated again in dH2O for 45 min. The digested flies were then washed with 70% ethanol and stored in 70% ethanol. The abdomen cuticles were cut from the rest of the digested fly using fine tweezers and an insulin syringe needle and placed in a drop of glycerol on a glass slide. The abdomens were then cut longitudinally on the dorsal side through all the tergites with the syringe. Some cuts were made between the tergites to ensure that the cuticle lay flat on the slide. The cuticles were then flattened with a coverslip. Photographs in the bright or dark field were taken on a Nikon SMZ18 stereomicroscope using Nikon DS-Ri2 digital camera, processed with ImageJ 1.50c4 and Fiji bundle 2.0.0-rc-46.

### Yeast two-hybrid assay

The yeast two-hybrid assay was performed as previously described [80]. Fragments of dCTCF cDNA were fused to either DNA-binding or activation domains of GAL4 using pGBT9 and pGAD424 vectors (Clontech, USA), respectively, with the primers listed in Additional File 4. Generated plasmids were transformed into *Saccharomyces cerevisiae* PJ69-4A strain (*MATa trp1–901 leu2*–*3*,*112 ura3*–*52 his3*–*200 gal4*∆ *gal80*∆ *LYS2*::*GAL1*-*HIS3 GAL2*-*ADE2 met2*::*GAL7*-*lacZ*) using the LiAc/SS-DNA/PEG method [81]. The cells were plated on medium lacking tryptophan and leucine. After three-day growth at 30ºC, the samples were streaked on selective medium lacking tryptophan, leucine, histidine, and adenine and incubated at 30ºC. Colony growth was assessed 3 days later. Each assay was repeated three times.

### Antibodies

Antibodies against the N-terminal domain (anti-dCTCF_N) of dCTCF (amino acids 1–287) and amino acids 657–818 of the C-terminal domain (anti-dCTCF_C) were produced in rabbits and purified by affinity purification on Aminolink resin (Thermo Fisher Scientific, USA) according to standard protocols. These antibodies were characterized in a previous study [34]. The other antibodies used were as follows: mouse monoclonal anti-HA antibodies, clone HA-7 (#H3663, Sigma, USA); mouse monoclonal anti-FLAG antibodies, clone M2 (F1804, Sigma, USA); mouse monoclonal anti-lamin Dm0, clone ADL84.12 (#ADL84.12, DSHB, USA); rat anti-CP190; anti-histone H3 (#39163, Active Motif, USA); anti-α-tubulin (#39527, Active Motif, USA).

### Fly extract preparation

#### Nuclear protein extract

Twenty adult flies were homogenized with a Dounce homogenizer (Wheaton) in buffer A (15 mM HEPES pH 7.6, 60 mM KCl, 15 mM NaCl, 10 mM EDTA, 0.5 mM EGTA, 0.5% NP40, 1mM DTT, 1 mM PMSF, and 1:500 Calbiochem Complete Protease inhibitor cocktail). The cells were collected by centrifugation at 3000 x *g* and resuspended in buffer B (20 mM Tris-HCl 7.4, 10 mM KCl, 10 mM MgCl2, 2 mM EDTA, 10% glycerol, 1% Triton X-100, 1mM DTT, 1 mM PMSF, and 1:500 Calbiochem Complete Protease inhibitor cocktail), sonicated 2 × 10 s at 5 W, and centrifuged at 3000 x *g* to separate the cytoplasm fraction from the nuclei. The nuclei were thrice washed with the same buffer, resuspended in buffer B, and 4x SDS-PAGE sample buffer with 2 M Urea and 300 mM NaCl was added to all the samples. The extracts then were boiled for 5 min at 100 °C, centrifuged for 5 min at 16,000 x *g*, and loaded on a 6% or 8% SDS–PAGE gels.

#### Total protein extract

Twenty adult flies were cooled in liquid nitrogen, homogenized for 30 s with a pestle in 200 µL of extraction buffer (20 mM HEPES, pH 7.5, 100 mM KCl, 5% glycerol, 10 mM EDTA, 1% NP-40, 1% sodium deoxycholate, 0.1% SDS, 1 mM DTT, 5 mM PMSF, and 1:100 Calbiochem Complete Protease Inhibitor Cocktails VII and V) and incubated on ice for 10 min. The suspension was sonicated in a Bioruptor (Diagenode, USA) for 3 min on setting H, 15 s ON/45 sec OFF. Then 4x SDS-PAGE sample buffer was added to the homogenate. Extracts were incubated for 10 min at 100 °C, centrifuged at 16,000 x *g* for 5 min, and loaded on a 6% or 8% SDS-PAGE gel.

#### Cellular Fractionation

Cellular fractionation was performed as described in [82], with some modifications. Sixty adult flies were homogenized with Potter and Dounce homogenizers (Wheaton) in 500 µL of ice-cold cytoplasm extraction buffer (CEB (50 mM HEPES-KOH pH 7.5, 10 mM NaCl, 1 mM EDTA pH8.0, 10% glycerol, 0.5% NP-40, 0.25% triton X-100, 1 mM DTT, and 1:100 Calbiochem Complete Protease Inhibitor Cocktails VII and V), filtered through a 70 μm cell strainer (Miltenyi Biotec, USA) and incubated on ice for 10 min. The homogenate was centrifuged at 3000 x *g* at 4 °C for 5 min.

The supernatant was collected in a fresh tube (cytoplasmic fraction), and the nuclear fraction was thrice washed with the same buffer. Then, the pellet was resuspended in 100 µL of ice-cold nuclear extraction buffer (NEB (10 mM Tris-HCl pH8.0, 200 (or 300) mM NaCl, 1 mM EDTA pH8.0, 0.5 mM EGTA pH8.0, and 1:100 Calbiochem Complete Protease Inhibitor Cocktails VII and V), incubated on ice for 10 min and centrifuged at 3000 x *g* at 4 °C for 5 min. The supernatant was collected in a fresh tube (nucleoplasmic fraction), and the chromatin pellet was thrice washed with the same buffer. Then, the pellet was resuspended in 100 µL of ice-cold chromatin extraction buffer (ChEB (500 mM Tris-HCl, 500 mM NaCl, and 1:100 Calbiochem Complete Protease Inhibitor Cocktails VII and V) and incubated on ice for 10 min. The solution (chromatin fraction) was sonicated in a Bioruptor (Diagenode, USA) for 5 min on setting H, 30 s ON/30 sec OFF and centrifuged at 16,000 x *g* at 4 °C for 10 min. The supernatant was collected in a fresh tube (chromatin fraction). Then 4x SDS–PAGE sample buffer was added to all the samples, and the extracts were boiled for 5 min at 100 °C, centrifuged for 5 min at 16,000 x *g*, and loaded on a 6% or 8% SDS–PAGE gel.

Protein samples were analyzed by immunoblot analysis. Proteins were detected using the ECL Plus Western Blotting substrate (Thermo Fisher Scientific, USA). Quantitative analysis of bands on immunoblots was performed using ImageLab 6.0.1 software (Bio-Rad) with ‘Relative quantity tool’.

### ChIP-Seq

Chromatin was prepared from two- to three-day-old adult flies. One gram of adult flies was ground in a mortar in liquid nitrogen and resuspended in 20 mL of buffer A (15 mM HEPES-KOH, pH 7.6, 60 mM KCl, 15 mM NaCl, 13 mM EDTA, 0.1 mM EGTA, 0.15 mM spermine, 0.5 mM spermidine, 0.5% NP-40, and 0.5 mM DTT) supplemented with 0.5 mM PMSF and Calbiochem Cocktail V. The suspension was then homogenized in a Potter and Dounce homogenizer with a tight pestle, filtered through a 100 μm Nylon Cell Strainer (Miltenyi Biotec, United States), and cross-linked with 1% formaldehyde for 15 min at room temperature. Cross-linking was stopped by adding glycine to a final concentration of 125 mM. The nuclei were washed with three 10-mL portions of wash buffer (15 mM HEPES-KOH, pH 7.6, 60 mM KCl, 15 mM NaCl, 1 mM EDTA, 0.1 mM EGTA, 0.1% NP-40, and protease inhibitors) and one 5-mL portion of nuclear lysis basic buffer (15 mM HEPES, pH 7.6, 140 mM NaCl, 1 mM EDTA, 0.1 mM EGTA, 1% Triton X-100, 0.5 mM DTT, 0.1% sodium deoxycholate, and protease inhibitors) and resuspended in 1 mL of nuclear lysis buffer (15 mM HEPES, pH 7.6, 140 mM NaCl, 1 mM EDTA, 0.1 mM EGTA, 1% Triton X-100, 0.5 mM DTT, 0.1% sodium deoxycholate, 0.5% SLS, 0.1% SDS, and protease inhibitors). The suspension was sonicated in a Covaris ME220 focused-ultrasonicator (40 alternating 15-s ON and 45-s OFF intervals, peak power 75, duty % factor 25), and 50-µL aliquots were used to test the extent of sonication and measure the DNA concentration. Debris was removed by centrifugation at 14,000 x *g*, 4 °C, for 10 min, and chromatin was pre-cleared with Protein A Dynabeads (Invitrogen, USA). Corresponding antibodies were incubated for 1 h at room temperature with 20 µL aliquots of Protein A (anti-CTCF C, 1:200) or G (anti-CP190, 1:200) Dynabeads (Invitrogen, USA) mixed with 200 µL of PBST. Then antibody–Dynabead complexes and anti-HA Magnetic beads (Pierce) were washed and equilibrated in nuclear lysis buffer. Chromatin samples containing 10–20 µg of DNA equivalent in 200 µL nuclear lysis buffer (2 µL aliquots of pre-cleared chromatin as input material) were incubated overnight at 4 °C with antibody–Dynabead complexes. After 3 rounds of washing with lysis buffer supplemented with 500 mM NaCl and TE buffer (10 mM Tris-HCl, pH 8; 1 mM EDTA), the DNA was eluted with elution buffer (50 mM Tris-HCl, pH 8.0; 1 mM EDTA, 1% SDS), the cross-links were reversed, and the precipitated DNA was extracted using a ChIP DNA Clean &Concentrator kit (Zymo Research, USA).

The ChIP-seq libraries were prepared with the NEBNext®_Ultra™_II DNA Library Prep kit per the manufacturer’s instructions. Amplified libraries were quantified using fluorometry with DS-11 (DeNovix, United States) and a Bioanalyzer 2100 (Agilent, United States). Diluted libraries were clustered on a pair-read flowcell and sequenced using a NovaSeq 6000 system (Illumina, United States). ChIP-seq analysis was performed for eight samples (CTCF^wt^-HA, CTCF^Δ132–170^-HA, CTCF^wt^, CTCF^Δ80–170^, CTCF^hN^, CP190, preimmune controls against HA, and rabbit antibodies). Two biological replicates were obtained for each sample. Reads were paired-end and were processed as described previously [34]. The main steps were as follows:

- Trimming Illumina adapters with cutadapt [83], parameters: -a “T{100}”, -m 20 --trim-n --minimum-length = 20 --pair-filter = any;
- Trimming low-quality ends with sickle (https://github.com/najoshi/sickle), parameters: -q 20 -l 20 –n;
- Alignment with bowtie2 [84], parameters: --no-discordant --no-mixed;
- Filtration of PCR duplicates and non-unique mapping with picard (https://broadinstitute.github.io/picard/) functions FilterSamReads and MarkDuplicates;
- Blacklist filtration with bedtools [85];
- Reproducible peak calling against corresponding preimmune controls with IDR pipeline (https://github.com/nboley/idr). A soft p-value threshold for MACS2 in IDR of 0.01 was used [86]. The IDR threshold was set to 0.05 for true replicates and 0.01 for pseudoreplicates.

All samples showed good reproducibility between biological replicates (the rescue and self-consistency ratios were below 2).

ChIP-seq coverage tracks (BedGraph) were obtained using deepTools [87] bamCoverage function, with a bin width of 50 bp and RPKM normalization (bam files from two replicates were preliminary merged). *De novo* motif discovery was performed using ChIPMunk [88, 89]. For motif discovery, the top 200 peaks per sample were narrowed to ± 200 bp around the summit, ChIPMunk was run in peak summit mode, and the motif length was set to 15. Genome-wide motif sites were identified using sarus (https://github.com/VorontsovIE/sarus) with a p-value threshold of 1 × 10^-4^. Heatmaps and coverage for peak sets were obtained with the deepTools functions computeMatrix and plotHeatmap [90].

Downstream analysis was performed in R statistical programming language, version 4.2.2. Peak annotation was performed using ChIPseeker [91] and GenomicRanges [92] packages; promoter segments were considered ± 200 bp from the TSS. The overlapping peaks were analyzed with ChIPpeakAnno [93] and visualized using the UpSetR package [94].

To compare CTCF^wt^-HA and CTCF^Δ132–170^-HA binding, we performed a linear regression of their signals (RPKM) in a combined set of their binding sites:

1 $$\eqalign{log10\left(RPKM(CTCF^{\Delta 132 - 170}HA)\right)\sim log10\left(RPKM( CTCF^{wt}HA) \right)\cr }$$

Then, we detected outliers as previously described [34] by calculating studentized regression residuals and finding those with the probability of arising from the normal distribution less than 0.05 (*p* < 0.05).

### Immunostaining of polytene chromosomes

*Drosophila* 3rd instar larvae were cultured at 18 °C under standard conditions. Polytene chromosome staining was performed as previously described [95]. The following primary antibodies were used: rat anti-CP190 at 1:300 dilution, anti-CTCF_C at 1:400 dilution, mouse anti-HA at 1:100 dilution. The secondary antibodies were Alexa Fluor 488 goat anti-rabbit 1:2000 and Alexa Fluor 555 goat anti-mouse 1:2000 (Invitrogen). The polytene chromosomes were co-stained with DAPI (AppliChem). Images were acquired with the Nikon Eclipse T*i* fluorescent microscope using Nikon DS-Qi2 digital camera, processed with ImageJ 1.50c4 and Fiji bundle 2.0.0-rc-46. Three to four independent stainings and 4–5 samples of polytene chromosomes were performed per transgenic line.

### Electronic supplementary material

Below is the link to the electronic supplementary material.

Additional file 1: Figures S1-S7.

Additional file 2. Comparative immunoblot analysis of cytoplasmic, nucleoplasmic, and chromatin fractions prepared from 2-day-old adult males of the *y*^*1*^*w*^*1118*^ and *wt-HA* lines.

Additional file 3. Immunoblot analysis of total extract and cytoplasmic, nucleoplasmic, chromatin fractions prepared from 2-day-old adult males of *y*^*1*^*w*^*1118*^*, wt-HA, Δ132-170, Δ80-170, hN* lines.

Additional file 4: Table S1. List of oligonucleotides used in the study.

## Data Availability

All data generated or analyzed during this study are included in this published article and its Additional files. Raw and processed ChIP-seq data were deposited in the NCBI Gene Expression Omnibus (GEO) under accession number GSE237742.
