# Supplementary material for: The N-terminal dimerization domains of human and Drosophila CTCF have similar functionality
Source: Epigenetics Chromatin. 2024 Apr 1;17:9. doi: 10.1186/s13072-024-00534-w (PMC10983669; doi:10.1186/s13072-024-00534-w)
Supplement: Supplementary file 1 — Additional file 1: Figures S1-S7. [file 13072_2024_534_MOESM1_ESM.pdf]

Additional file 1: Figures S1-S7

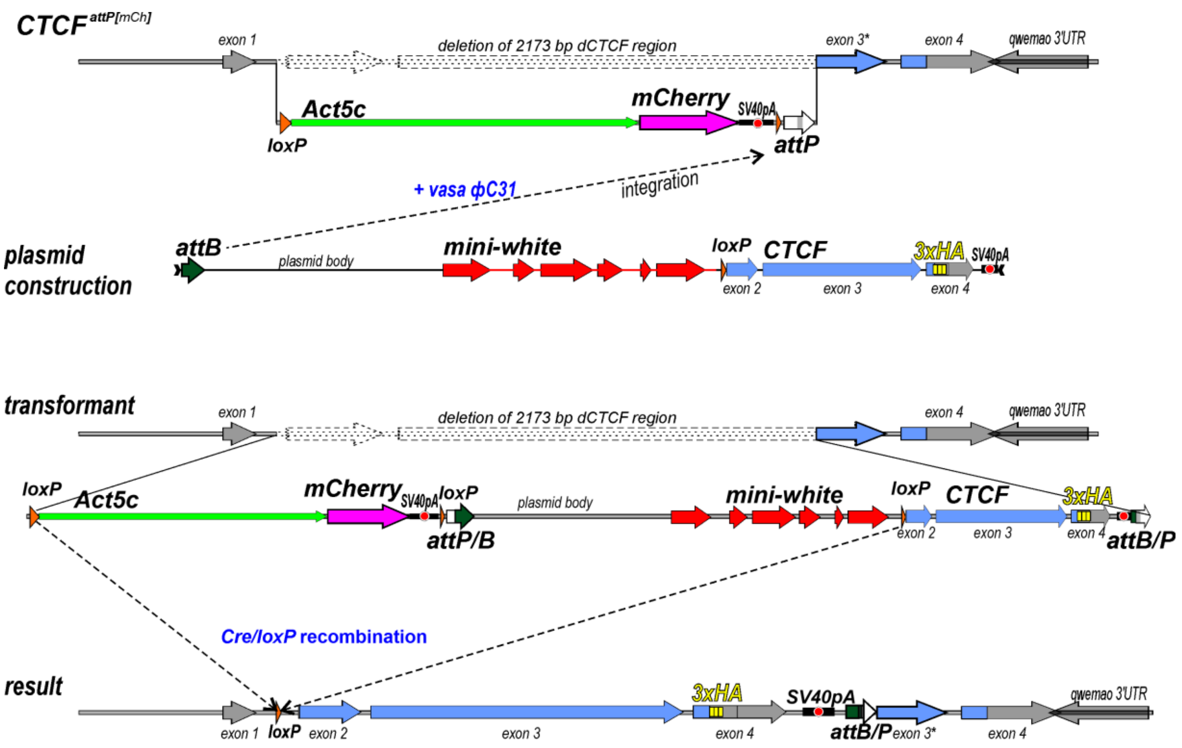

Figure S1. Strategy for generating the *CTCF* alleles. In the $CTCF^{attP(mCh)}$ platform, the 2,173-bp *CTCF* gene, from the first intron to the end of the third exon (3L:7352322-7358209, r6.52), was replaced with the *attP-Act5c-mCherry*. The deleted part of *CTCF* is shown as light gray arrows with a gray dotted outline. The *dCTCF*-coding and untranslated regions (UTRs) are shown as blue and gray horizontal arrows, respectively. The surrounding genome regions are indicated as gray lines. The *mCherry* reporter (magenta arrow) is controlled by the *Act5C* promoter, which is depicted in green. The *attP* and *loxP* sites used for genome manipulations are shown as white and orange arrows, respectively. The construct for the replacement contains the *mini-white* reporter, a *loxP* site, and the CTCF-coding region fused with a 3×HA tag. The φC31-mediated recombination results in the integration of a complete construct, including the plasmid DNA, into the $CTCF^{attP(mCh)}$ platform. Cre-mediated recombination between the *loxP* sites generates the deletion of all sequences except the *CTCF* gene and one *loxP* site in the first intron.

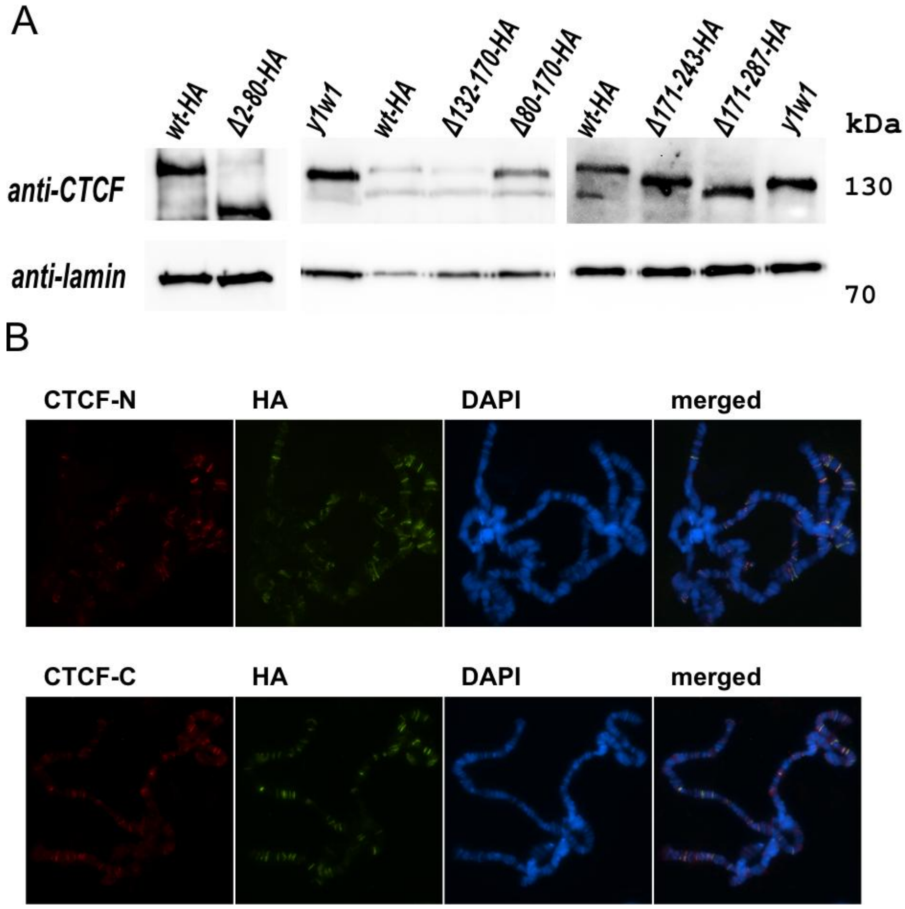

Figure S2. (A) Immunoblot analysis (6% SDS PAGE) of protein extracts prepared from 2-day-old adult males of the *dCTCF*^{wt}*-HA (wt-HA), dCTCF*$^{\Delta 2\text{-}80}$*-HA (Δ2-80-HA), dCTCF*$^{\Delta 132–170}$*-HA (Δ132–170-HA), dCTCF*$^{\Delta 80–170}$*-HA (Δ80–170-HA), dCTCF*$^{\Delta 80–125}$*-HA (Δ80–125-HA), dCTCF*$^{\Delta 171\text{-}243}$*-HA (Δ171-243-HA),* and *dCTCF*$^{\Delta 171\text{-}287}$*-HA (Δ171-287-HA)* lines with antibodies against dCTCF_C and lamin (internal control) antibodies. (B) Distribution of dCTCF^{WT}-HA in the polytene chromosomes of third instar female larvae. The panels show the results of immunostaining with rabbit anti-dCTCF_C, rabbit anti-dCTCF_N, and mouse anti-HA antibodies. DNA was stained with DAPI (blue).

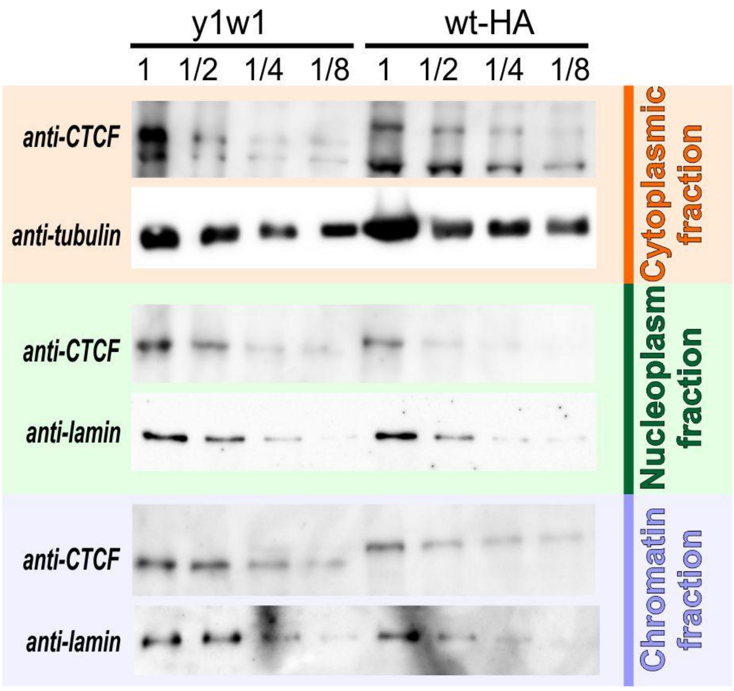

Figure S3. Immunoblot analysis of cytoplasmic, nucleoplasmic, and chromatin fractions prepared from 2-day-old adult males of the $y^{1}w^{1118}$ (y1w1) and $dCTCF^{wt}$-*HA* (wt-HA) lines. The cytoplasmic, nucleoplasmic, and chromatin fractions were prepared as described in the Materials and Methods section. Samples were titrated at two-fold dilutions (‘1’, ‘1/2’, ‘1/4’, ‘1/8’). Immunoblot analysis was performed using antibodies against the N-terminal region of dCTCF (anti-dCTCF_N) and control antibodies against α-tubulin (cytoplasmic marker), lamin Dm0 (nuclear marker).

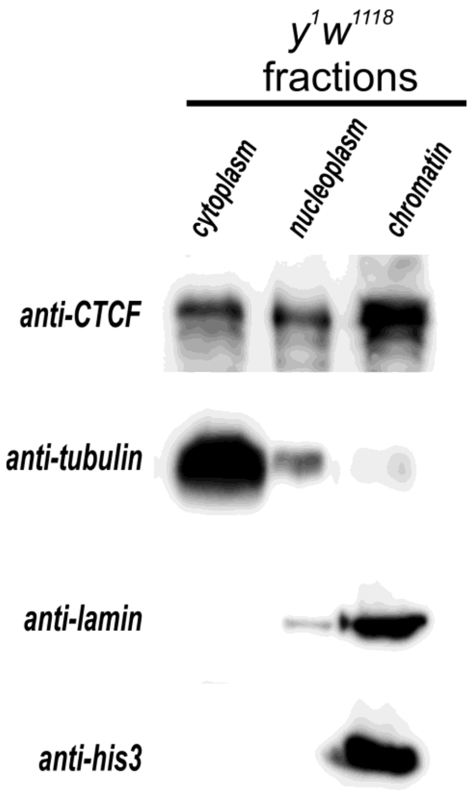

Figure S4. Immunoblot analysis of cytoplasmic, nucleoplasmic, and chromatin fractions prepared from 2-day-old adult males of the *y^1w^{1118}* line. The cytoplasmic, nucleoplasmic, and chromatin fractions were prepared as described in the Materials and Methods section. Immunoblot analysis was performed using antibodies against the N-terminal region of dCTCF (anti-dCTCF_N) and control antibodies against α-tubulin (cytoplasmic marker), lamin Dm0 (nuclear marker), and histone H3 (his3, chromatin marker).

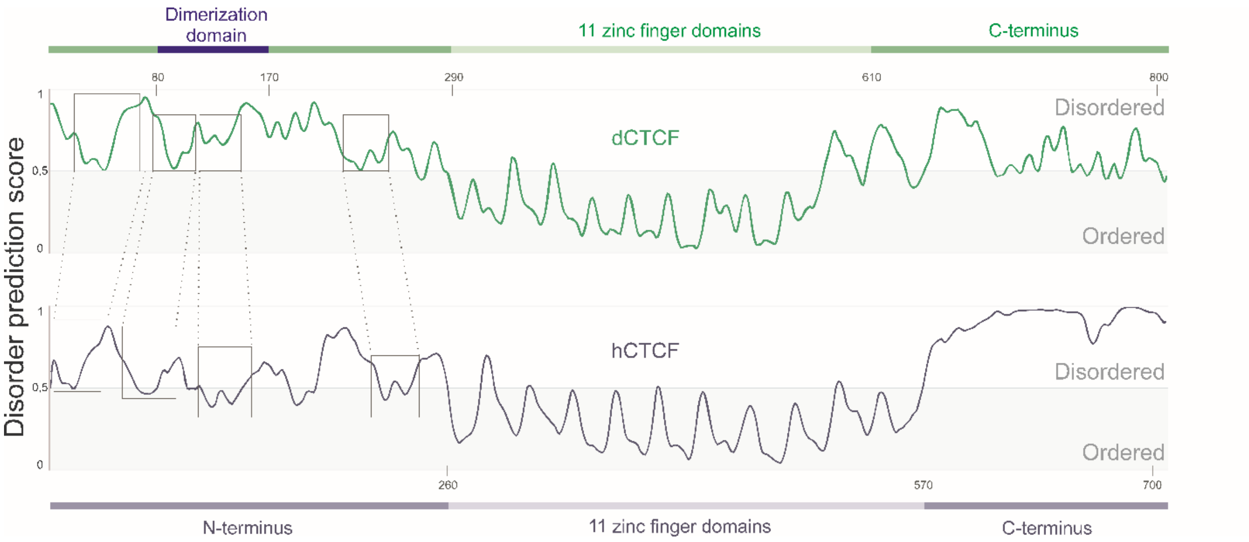

Figure S5. Disorder profiles of dCTCF (green) and hCTCF (dark grey) amino acid sequences predicted with IUPRED3 [1]**.** The x-axis of the plot represents the amino acid positions along the protein sequence. The domain structure is depicted schematically. Regions with high disorder propensity are shown as peaks or peaks with a plateau, indicating that those regions are likely to be intrinsically disordered. In contrast, regions below the baseline are predicted to be structured (as seen in Zinc finger domains). Black squares indicate N-terminal regions with similar intrinsically disordered patterns demonstrating the putative structural resemblance of the proteins despite the lack of sequence homology.

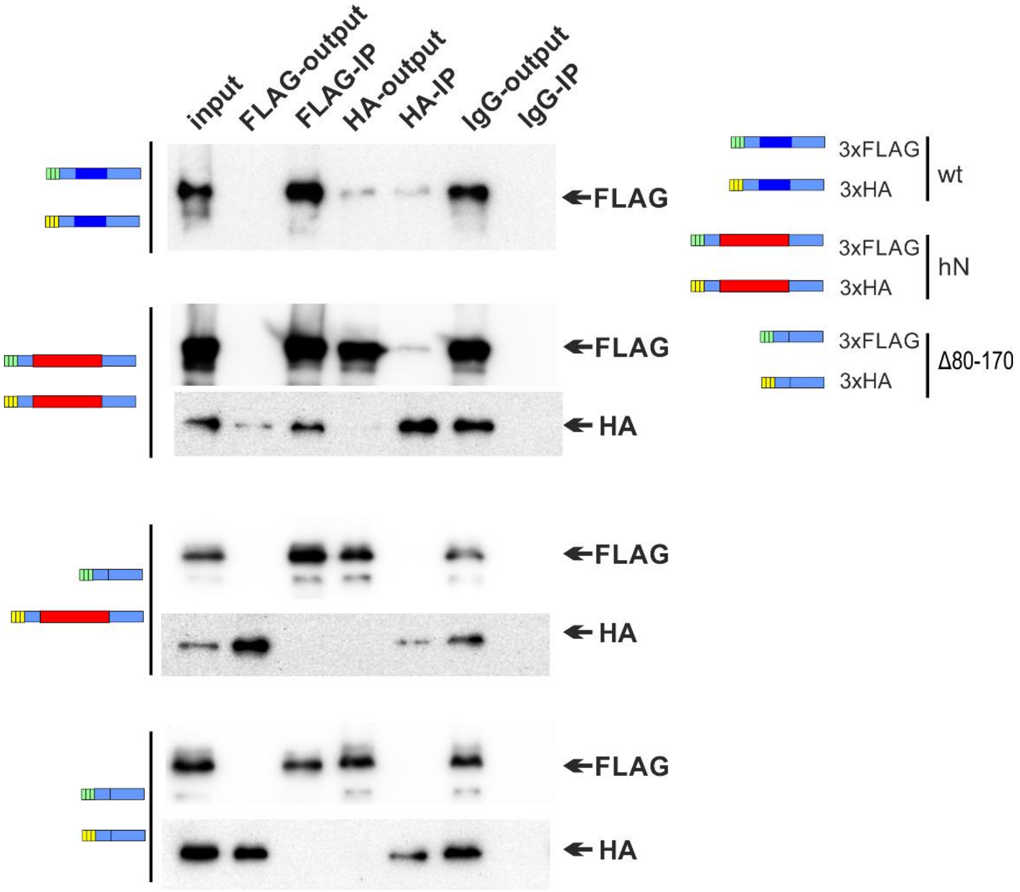

Figure S6. Co-immunoprecipitation of dCTCF N-terminal domains (wild type (wt), deletion of 80-170 aa (Δ80–170), insertion of human CTCF N-terminus into dCTCF with deleted 80–170 aa region (hN)). Total extracts from *Drosophila* S2 cells co-transfected with N-terminal domains of dCTCF tagged with 3xHA and 3xFLAG epitopes were immunoprecipitated with antibodies against HA, FLAG or non-specific IgG as a negative control, and the immunoprecipitates were analyzed by Western blotting for the presence of FLAG- and HA-tagged proteins.

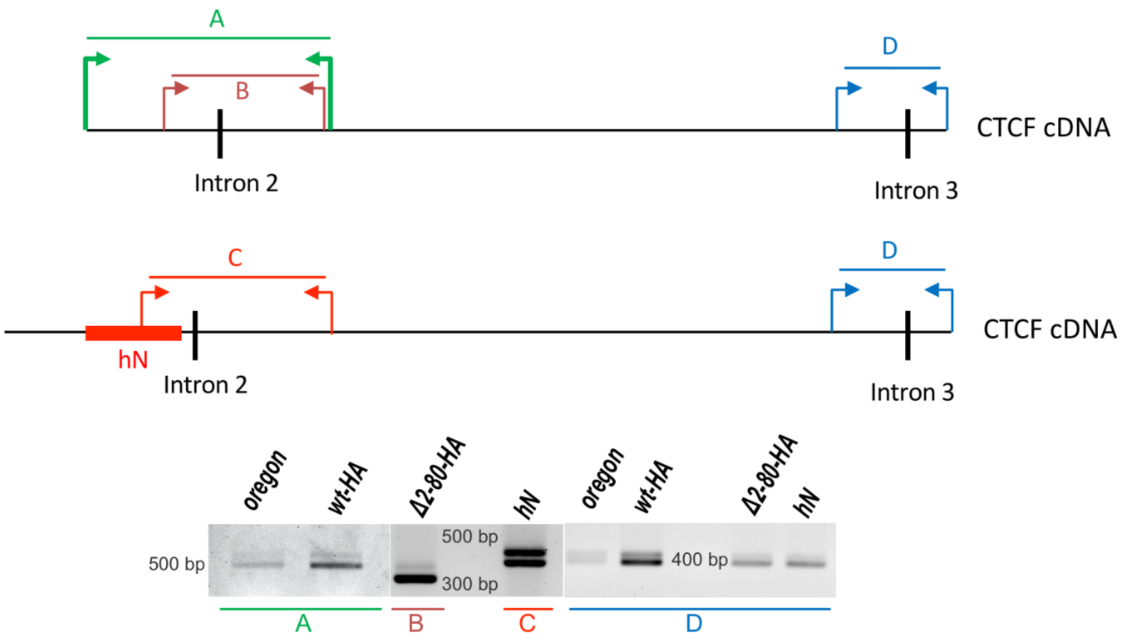

Figure S7. **Analysis of splicing variants with PCR of cDNA from adult flies** (lines *oregon, wt-HA, Δ2-80-HA, hN*). Pairs of used primers (A, B and C to specifically assess splicing of intron 2 and D for intron 3) are schematically indicated above and available upon request. **hN** spliced (lower band) and unspliced (upper band) variants are present in equal amounts, whereas oregon, wt-HA, Δ2-80-HA have predominantly already spliced transcript variants.

References

1. Erdos G, Pajkos M, Dosztányi Z (2021) IUPred3: Prediction of protein disorder enhanced with unambiguous experimental annotation and visualization of evolutionary conservation. Nucleic Acids Res 49:W297–W303. https://doi.org/10.1093/nar/gkab408
