# Supplementary material for: The N-terminal dimerization domains of human and Drosophila CTCF have similar functionality
Source: Epigenetics Chromatin. 2024 Apr 1;17:9. doi: 10.1186/s13072-024-00534-w (PMC10983669; doi:10.1186/s13072-024-00534-w)
Supplement: Supplementary file 2 — Additional file 2. Comparative immunoblot analysis of cytoplasmic, nucleoplasmic, and chromatin fractions prepared from 2-day-old adult males of the y1w1118 and wt-HA lines. [file 13072_2024_534_MOESM2_ESM.pdf]

Comparative immunoblot analysis of cytoplasmic, nucleoplasmic, and chromatin fractions prepared from 2-day-old adult males of the *y^{1} w^{1118}* (y1w1) and *$dCTCF^{wt}$-HA* (wt-HA) lines

Cytoplasmic extract

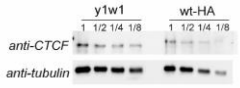

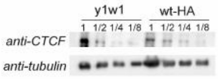

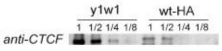

	a-CTCF	a-tubulin	ratio
y1w1	1	1	1
y1w1 1/2	0.444227	0.753819	0.5893019
y1w1 1/4	0.324511	0.443724	0.7313352
y1w1 1/8	0.234832	0.256362	0.9160172
wt-HA	0.518943	0.751781	0.6902848
wt-HA 1/2	0.189041	0.436122	0.433459
wt-HA 1/4	0.070104	0.217877	0.3217595
wt-HA 1/8	0.033413	0.086246	0.3874151

	a-CTCF	a-tubulin	ratio
y1w1	1	1	1
y1w1 1/2	0.20373	0.734247	0.277468
y1w1 1/4	0.033663	0.543773	0.061906
y1w1 1/8	0.067262	0.563319	0.119403
wt-HA	0.41555	1.256703	0.330667
wt-HA 1/2	0.109689	0.722232	0.151875
wt-HA 1/4	0.052723	0.617942	0.08532
wt-HA 1/8	0.029182	0.583701	0.049995

	a-CTCF
y1w1	1
y1w1 1/2	0.643057
y1w1 1/4	0.176916
y1w1 1/8	0.053122
wt-HA	0.185183
wt-HA 1/2	0.125211
wt-HA 1/4	0.026472
wt-HA 1/8	0.002141

	a-CTCF		a-tubulin	
	mean	SD	mean	SD
y1w1	1		1	
y1w1 1/2	0.430338	0.219992571	0.744033	0.0138395
y1w1 1/4	0.1783633	0.145429402	0.4937485	0.0707453
y1w1 1/8	0.1184053	0.101076018	0.4098405	0.2170514
wt-HA	0.3732253	0.170858033	1.004242	0.3570338
wt-HA 1/2	0.1413137	0.042055403	0.579177	0.2023103
wt-HA 1/4	0.0497663	0.021965752	0.4179095	0.2828887
wt-HA 1/8	0.0215787	0.016965922	0.3349735	0.3517538

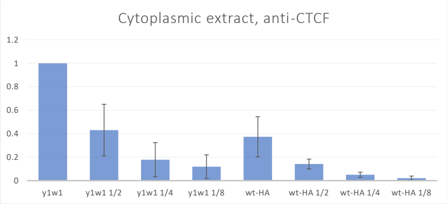

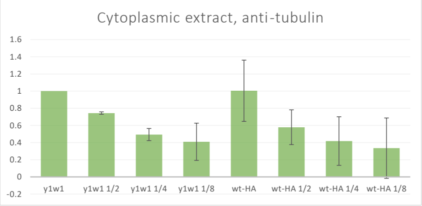

Nucleoplasmic extract

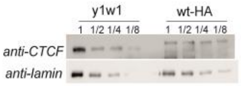

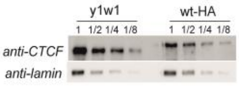

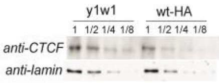

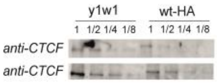

	a-CTCF	a-lamin	ratio
y1w1	1	1	1
y1w1 1/2	0.182827	0.277981	0.657696
y1w1 1/4	0.17482	0.093104	1.8776852
y1w1 1/8	0.044257	0.007807	5.6688869
wt-HA	0.141154	0.419679	0.336338
wt-HA 1/2	0.148681	0.272157	0.546306
wt-HA 1/4	0.121391	0.0938	1.2941471
wt-HA 1/8	0.097089	0.03911	2.4824597

	a-CTCF	a-lamin	ratio
y1w1	1	1	1
y1w1 1/2	0.346019	0.231505	1.49465
y1w1 1/4	0.20526	0.074171	2.767389
y1w1 1/8	0.068734	0.019311	3.559319
wt-HA	0.268775	0.901267	0.298219
wt-HA 1/2	0.147032	0.346561	0.42426
wt-HA 1/4	0.051482	0.076106	0.676451
wt-HA 1/8	0.020068	0.024764	0.81037

	a-CTCF	a-lamin	ratio
y1w1	1	1	1
y1w1 1/2	0.267396	0.485389412	0.55089
y1w1 1/4	0.129119	0.085095978	1.517334
y1w1 1/8	0.069261	0.017942291	3.860209
wt-HA	0.231314	1.102212984	0.209863
wt-HA 1/2	0.117959	0.317765008	0.371215
wt-HA 1/4	0.043483	0.034050617	1.277011
wt-HA 1/8	0.03954	0.014213229	2.781915

	a-CTCF	a-CTCF
y1w1	1	1
y1w1 1/2	0.704664	0.637494
y1w1 1/4	0.15764	0.212082
y1w1 1/8	0.140925	0.209202
wt-HA	0.489801	0.536487
wt-HA 1/2	0.168552	0.155231
wt-HA 1/4	0.114167	0.064165
wt-HA 1/8	0.070678	0.047193

	a-CTCF		a-lamin	
	mean	SD	mean	SD
y1w1	1		1	
y1w1 1/2	0.42768	0.230789147	0.3316251	0.1351762
y1w1 1/4	0.1757842	0.034256881	0.0841237	0.0095039
y1w1 1/8	0.1064758	0.06786012	0.0150201	0.0062841
wt-HA	0.3335062	0.171217707	0.8077197	0.3507513
wt-HA 1/2	0.147491	0.018555586	0.312161	0.0375172
wt-HA 1/4	0.0789376	0.036305888	0.0679855	0.0306913
wt-HA 1/8	0.0549136	0.02973824	0.0260291	0.0124965

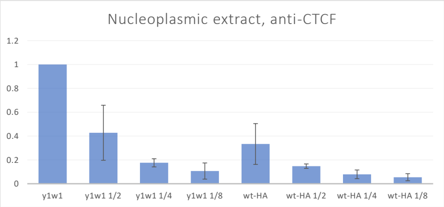

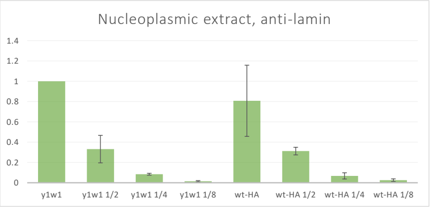

Chromatin extract

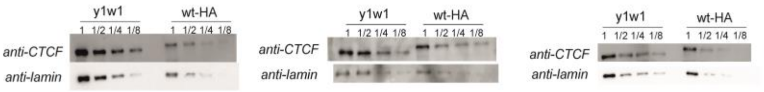

	a-CTCF	a-lamin	ratio
y1w1	1	1	1
y1w1 1/2	0.439322	0.511586	0.8587452
y1w1 1/4	0.354168	0.234305	1.5115683
y1w1 1/8	0.128004	0.121688	1.0519032
wt-HA	0.298041	0.501129	0.5947391
wt-HA 1/2	0.065594	0.104349	0.6286021
wt-HA 1/4	0.021261	0.061699	0.3445923
wt-HA 1/8	0.009369	0.001165	8.0420601

	a-CTCF	a-lamin	ratio
y1w1	1	1	1
y1w1 1/2	0.809668	0.780126	1.037868
y1w1 1/4	0.360795	0.434196	0.83095
y1w1 1/8	0.168115	0.050236	3.346504
wt-HA	0.664373	0.816533	0.813651
wt-HA 1/2	0.237571	0.318886	0.745003
wt-HA 1/4	0.213634	0.126975	1.682489
wt-HA 1/8	0.100242	0.045816	2.187926

	a-CTCF	a-lamin	ratio
y1w1	1	1	1
y1w1 1/2	0.403848	0.399112	1.011866
y1w1 1/4	0.288704	0.205745	1.403213
y1w1 1/8	0.150412	0.052179	2.882616
wt-HA	0.602413	0.896558	0.671917
wt-HA 1/2	0.202708	0.109642	1.848817
wt-HA 1/4	0.074478	0.069178	1.076614
wt-HA 1/8	0.01383	0.001608	8.600746

	a-CTCF		a-lamin	
	mean	SD	mean	SD
y1w1	1		1	
y1w1 1/2	0.550946	0.224760775	0.563608	0.1957617
y1w1 1/4	0.3345557	0.039846716	0.2914153	0.1244735
y1w1 1/8	0.1488437	0.020101439	0.074701	0.0407035
wt-HA	0.521609	0.196078424	0.7380733	0.2090645
wt-HA 1/2	0.1686243	0.090913664	0.1776257	0.1223637
wt-HA 1/4	0.1031243	0.099334295	0.0859507	0.0357244
wt-HA 1/8	0.041147	0.051226355	0.0161963	0.0256523

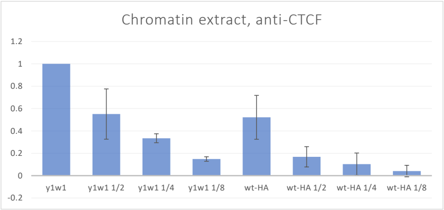

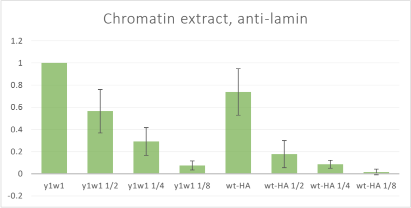

Comparison

	mean ratio			sd ratio		
	cytoplasm	nucleoplasm	chromatin	cytoplasm	nucleoplasm	chromatin
y1w1	1	1	1			
wt-HA	0.5104758	0.281473441	0.6934359	0.2542883	0.06487895	0.11103109

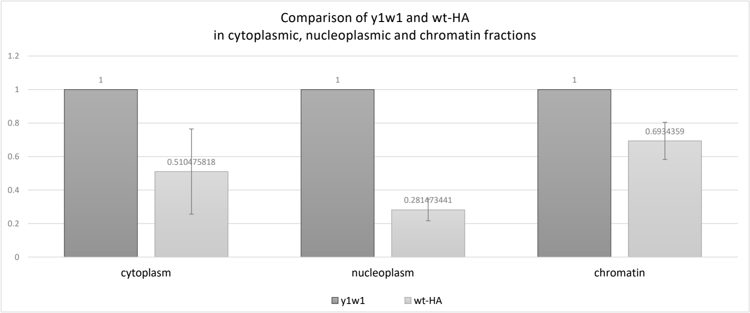

Samples were titrated at two-fold dilutions (‘1’, ‘1/2’, ‘1/4’, '1/8').
Immunoblot analysis was performed using antibodies against the N-terminal region of dCTCF (anti-dCTCF_N) and control antibodies against α-tubulin (cytoplasmic marker), lamin Dm0 (nuclear marker).
