# Supplementary material for: The N-terminal dimerization domains of human and Drosophila CTCF have similar functionality
Source: Epigenetics Chromatin. 2024 Apr 1;17:9. doi: 10.1186/s13072-024-00534-w (PMC10983669; doi:10.1186/s13072-024-00534-w)
Supplement: Supplementary file 3 — Additional file 3. Immunoblot analysis of total extract and cytoplasmic, nucleoplasmic, chromatin fractions prepared from 2-day-old adult males of y1w1118, wt-HA, Δ132-170, Δ80-170, hN lines. [file 13072_2024_534_MOESM3_ESM.pdf]

Immunoblot analysis of total extract and cytoplasmic, nucleoplasmic, chromatin fractions prepared from two-day-old adult males of y^1w^{1118} (y1w1), dCTCFwt-HA (wt-HA), dCTCFΔ80–170-HA (Δ80–170-HA), dCTCFΔ132–170-HA (Δ132–170-HA), and dCTCFhN (hN).

Total extract

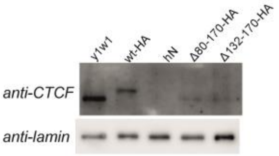

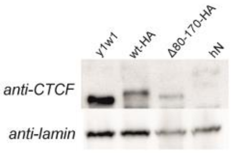

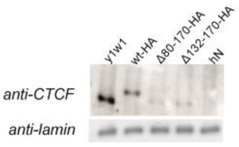

	a-CTCF	a-lamin	ratio
y1w1	1	1	1
wt-HA	0.417534	1.193518	0.349835
hN		1.513725	0
Δ80-170-HA	0.135118	1.29047	0.104704
Δ132-170-HA	0.180456	2.163253	0.083419

	a-CTCF	a-lamin	ratio
y1w1	1	1	1
wt-HA	0.211284	0.882318	0.239465
hN	0.018915	0.467508	0.040459
Δ80-170-HA	0.067774	0.329514	0.205679

	a-CTCF	a-lamin	ratio
y1w1	1	1	1
wt-HA	0.52661	0.945717	0.556837
hN		1.063185	0
Δ80-170-HA	0.108821	1.068352	0.101858
Δ132-170-HA	0.051874	1.106947	0.046863

	a-CTCF		a-lamin		ratio	
	mean	SD	mean	SD	mean	SD
y1w1	1		1		1	
wt-HA	0.385143	0.160139	1.007184	0.164454	0.38204534	0.161119
hN	0.018915		1.014806	0.524784	0.0134864	
Δ80-170-HA	0.103904	0.03394	0.896112	0.503099	0.13741384	0.059136
Δ132-170-HA	0.116165	0.090921	1.6351	0.746921	0.06514067	0.025849

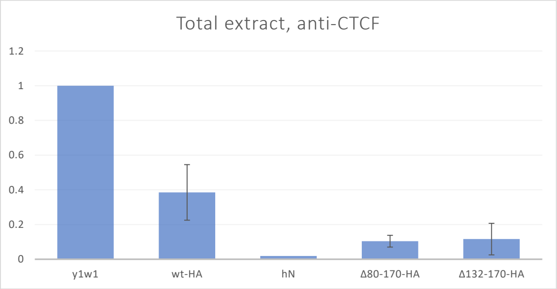

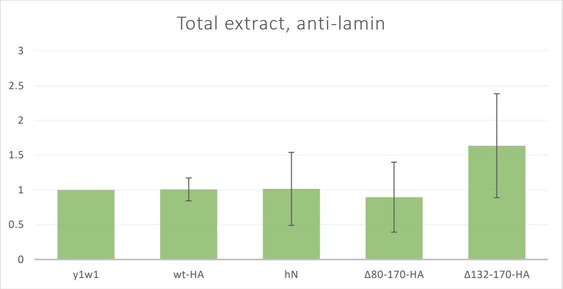

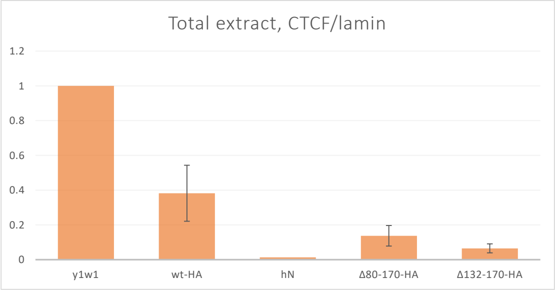

Cytoplasmic extract

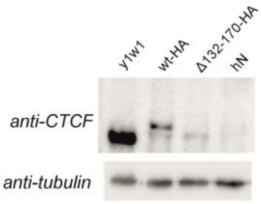

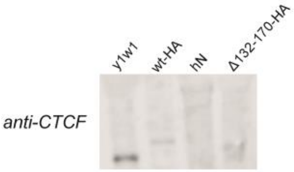

	a-CTCF	a-tubulin	ratio
y1w1	1	1	1
wt-HA	0.266472	1.200114	0.366723
hN		1.206194	
Δ132-170-HA	0.056297	1.50729	0.292601

	a-CTCF
y1w1	1
wt-HA	0.440109
hN	
Δ132-170-HA	0.441035

	mean a-CTCF
y1w1	1
wt-HA	0.353290389
hN	
Δ132-170-HA	0.24866602

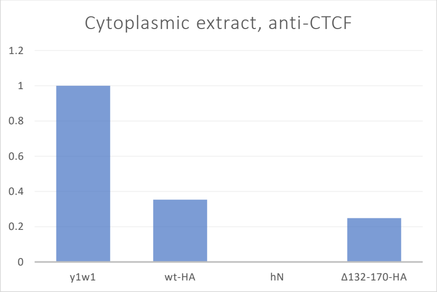

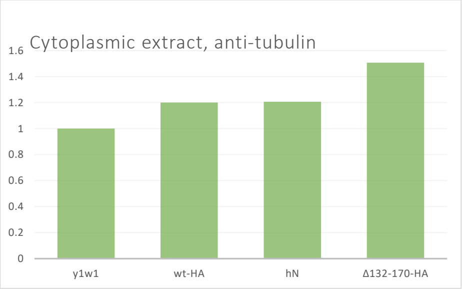

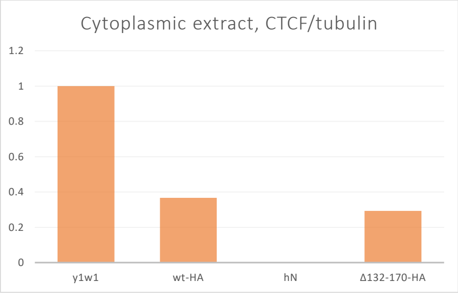

Nucleoplasmic extract

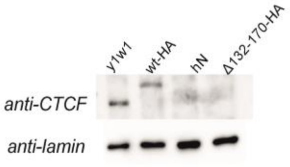

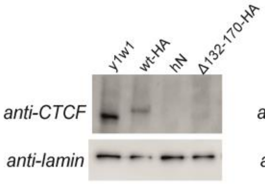

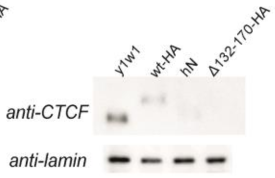

	a-ctcf	a-lamin	ratio
y1w1	1	1	1
wt-HA	0.625281	1.507173	0.41487
hN		2.042857	
Δ132-170-HA		1.882478	

	a-ctcf	a-lamin	ratio
y1w1	1	1	1
wt-HA	0.300476	0.427426	0.70299
hN		1.017673	
Δ132-170-HA		0.692333	

	a-ctcf	a-lamin	ratio
y1w1	1	1	1
wt-HA	0.258133	0.597305	0.432163
hN		0.957027	
Δ132-170-HA		0.905273	

	a-CTCF		a-lamin		ratio	
	mean	SD	mean	SD	mean	SD
y1w1	1		1		1	
wt-HA	0.39463	0.200868	0.843968	0.580599	0.51667414	0.161585
hN			1.339186	0.610151		
Δ132-170-HA			1.160028	0.634655		

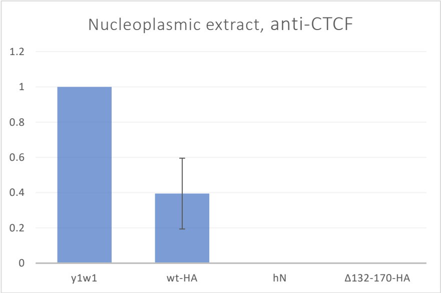

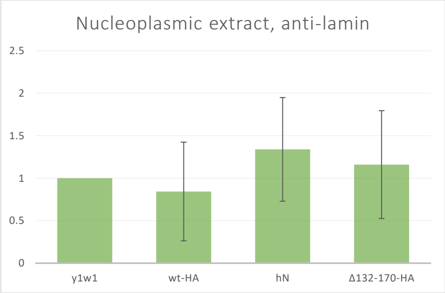

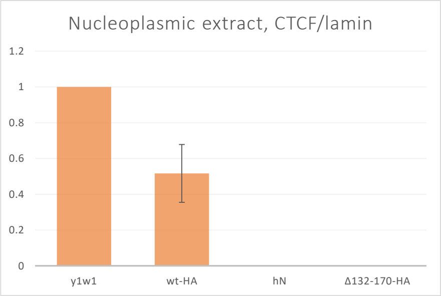

Chromatin extract

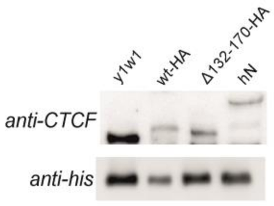

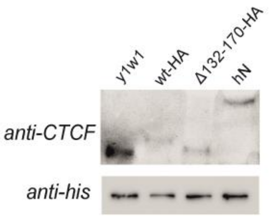

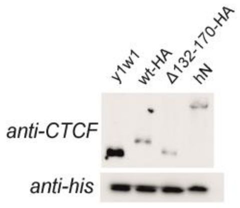

	a-ctcf	a-histone	ratio
y1w1	1	1	1
wt-HA	0.124998	0.390532	0.320071
Δ132-170-HA	0.230332	0.837625	0.274982
hN	0.141635	0.876036	0.161677

	a-ctcf	a-histone	ratio
y1w1	1	1	1
wt-HA	0.112525	0.556886	0.202061
Δ132-170-HA	0.202752	0.984865	0.205868
hN	0.515182	1.256353	0.410062

	a-ctcf	a-histone	ratio
y1w1	1	1	1
wt-HA	0.280428	0.917717	0.305571
Δ132-170-HA	0.190571	0.927403	0.205489
hN	0.289128	0.761128	0.379868

	a-CTCF		a-histone		ratio	
	mean	SD	mean	SD	mean	SD
y1w1	1		1		1	
wt-HA	0.17265	0.093546	0.621712	0.269505	0.27590117	0.064357
Δ132-170-HA	0.207885	0.020371	0.916631	0.074209	0.22877964	0.040013
hN	0.315315	0.188145	0.964506	0.259195	0.31720214	0.135532

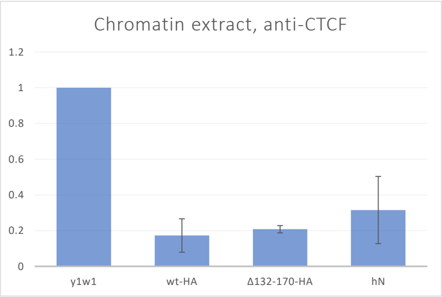

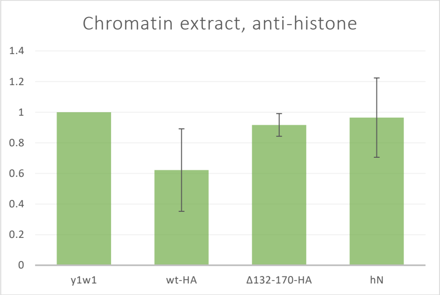

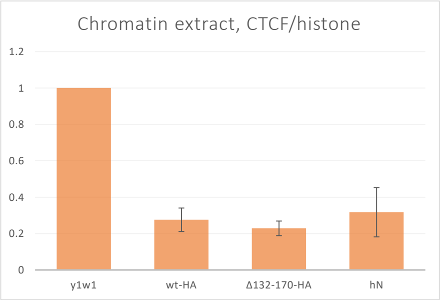

Blots were stained with anti-dCTCF_C antibodies and control antibodies against tubulin (cytoplasmic marker), lamin (nuclear marker), and histone H3 (his3, chromatin marker).
