# Supplementary material for: The N-terminal dimerization domains of human and Drosophila CTCF have similar functionality
Source: Epigenetics Chromatin. 2024 Apr 1;17:9. doi: 10.1186/s13072-024-00534-w (PMC10983669; doi:10.1186/s13072-024-00534-w)
Supplement: Supplementary file 4 — Additional file 4: Table S1. List of oligonucleotides used in the study. [file 13072_2024_534_MOESM4_ESM.pdf]

Additional file 4

Table S1. List of oligonucleotides used in the study

Used for		**Primer**	**Sequence 5'-3'**
Yeast two-hybrid assay		N_dCTCFH1_d	ataggatcctgccaaggaggacaaaaaaggac
		dCTCF_90rev	atagtcgacctcgtcatctatgaaatacttatcctc
		dCTCF_132Sr	ccggtcgacgatggatttattggtggct
		dCTCF_170Sr	tgggtcgaccgattgcttcgccttggct
		dCTCFdel80dir	aggaggagacagccaccaataaatccat
		dCTCFdel125rev	gtggctgtctcctcctcctcctccg
		dCTCF_288rev	gtggctgtctcctcctcctcctccg
CTCF replacement	To vector elements	lox_dir	gtatcgccggggatctat
		white_exon5_d	tgaccaacatgacctttc
		pAc_rev	tcactgcattctagttgtggt
		tSV40RT2	agccataccacatttgtagag
	Δ2-80	ctcfDel2-80d	taggaatggacgaggataagtatttcatag
		ctcfDel2-80r	tcctcgtccattcctatggacaaattgg
	Δ80-125	ctcfDel_80_r	ggtggctgtctcctcctcctcctccgc
		ctcfDel_125_d	gaggaggagacagccaccaataaatgtgag
	Δ132-170	Ctcf_del170_d	taagccatcgcgatgccaccgccaccggc

		Ctcf_del133_r	gatggcttaaaaagagtcgagcaagt
	Δ171-243	ctcfDel243_d	aagcaatcggaggctgaggtctacgaatttg
		ctcfDel243_171r	ctcagcctccgattgcttcgccttggc
	Δ171-287	ctcfDel287_d	aagcaatcgaatgcatcgggacataagtac
		ctcfDel287_171r	cgatgcattcgattgcttcgccttggc
	Human CTCF 2-265	dd_humCTCF_1r	atcaccttcctcctcctcctcctccgc
		ddC_h265d	gtaaagaagacaacagccaccaataaatgtgag
		h265ddC_r	ggtggctgttgtcttctttacacctttc
		humCTCFdd_dir	gaggaggaggaaggtgatgcagtcgaag
Sequencing	Genomic region	Greg_dir	ccgcttacgcagcccaaatcc
		Greg_rev	tgatgcactgcacggcagg
		GregSeq_r	actaatagccgagaatttac
	CTCF CDS	CTCF189r	ttctcgagttaaggcctgcccctgggtg
		CTCF_zf3d	ccaaatttcactgcaactactgcccgacga
		CTCF547r	cggggtaccgtccaggtggatgagcatg
